# Genetic Diversity of Porcine Reproductive and Respiratory Syndrome Virus (PRRSV) From 1996 to 2017 in China

**DOI:** 10.3389/fmicb.2020.00618

**Published:** 2020-04-24

**Authors:** Yifeng Jiang, Guoxin Li, Lingxue Yu, Liwei Li, Yujiao Zhang, Yanjun Zhou, Wu Tong, Changlong Liu, Fei Gao, Guangzhi Tong

**Affiliations:** ^1^Research Team on Porcine Viral Reproductive Disorder Syndrome, Shanghai Veterinary Research Institute, Chinese Academy of Agricultural Sciences, Shanghai, China; ^2^Jiangsu Co-innovation Center for the Prevention and Control of Important Animal Infectious Disease and Zoonosis, Yangzhou University, Yangzhou, China

**Keywords:** genetic diversity, *porcine reproductive and respiratory syndrome virus* (PRRSV), recombination, phylogenetic trees, evolution

## Abstract

Porcine reproductive and respiratory syndrome (PRRS) is one of the most devastating diseases of the global swine industry. The causative agent *porcine reproductive and respiratory syndrome virus* (PRRSV) was first isolated in China in 1996 and has evolved quickly during the last two decades. To fully understand virus diversity, epidemic situation in the field, and make future predictions, a total of 365 PRRSV strains were used for evolution and genome analysis in which 353 strains were isolated from mainland China. The results showed that high diversity was found among PRRSV isolates. Total PRRSV isolates could be divided into eight subgroups. Among these subgroups strains, Original HP-PRRSV, NADC30-like, and Intermediate PRRSV were the major epidemic PRRSV strains circling in the field and would play a major role in PRRS epidemic in the future. Deletions, insertions, and recombinations have occurred frequently in the PRRSV genome. Deletions were the main driving force of viral evolution before 2006 and may also contribute further to the virus' evolution in a relatively closed or low strain diversity circumstance. The recombinant strains could be divided into three groups: the Inner group, Extensional group, and Propagating group. The evolutionary directions of the isolates in the Extensional and Propagating groups have changed, and the routes of recombination in the Propagating group were analyzed and sorted into three types. The increases in recombinant strains and high rates of recombination in recent years indicate that recombination has played a very important role in the virus' evolution. Isolates, which incorporate the advantages of their parental strains, will influence PRRSV evolution and make adverse effects on PRRS control in the future.

## Introduction

Porcine reproductive and respiratory syndrome (PRRS) is one of the most devastating diseases of the global swine industry (Benfield et al., 1992; Wensvoort et al., 1992; Nelsen et al., 1999; Neumann et al., 2005; An et al., 2010; Zhou and Yang, 2010; Jantafong et al., 2015). The causative agent, *porcine reproductive and respiratory syndrome virus* (PRRSV), is an enveloped single-stranded positive-sense RNA virus belonging to the family *Arteriviridae* in the order *Nidovirales* (Benfield et al., 1992; Snijder and Meulenberg, 1998). The PRRSV genome is approximately 15 kb in length, and contains at least 10 open reading frames (ORFs), a short 5′ untranslated region (Lassalle et al., 2004), and a poly(A) tail at the 3′ terminus. ORF1a and ORF1ab encode the replication-related polymerase proteins and are processed into at least 13 non-structural proteins by self-cleavage, and the other ORFs encode eight structural proteins (Snijder et al., 1994; Bautista et al., 1996; van Dinten et al., 1996; Meulenberg et al., 1997; Firth et al., 2011; Johnson et al., 2011). PRRSV exists as two major genotypes, the European prototype (EU-type, type 1), known as the Lelystad virus (LV), and the North American prototype (NA-type, type 2), known as VR-2332 (Benfield et al., 1992; Wensvoort et al., 1992). Strains LV and VR-2332 share about 55–70% nucleotide identity at the genome level and about 50–80% amino acid similarity (Suarez et al., 1996; Nelsen et al., 1999).

The first Chinese PRRSV strain CH-1a was isolated from an aborted pig fetus in 1996 (Valicek et al., 1997). In 2006, the highly pathogenic PRRSV (HP-PRRSV) with a characteristic 1 + 29 amino acid deletion in the NSP2 coding region first emerged in Jiangxi Province and then spread rapidly to other provinces, causing huge economic losses and became the major circulating strain in pig farms in China (Tong et al., 2007; Guo et al., 2012; Liu et al., 2013; Xie et al., 2013). The virulence of HP-PRRSV was enhanced and characterized by high fever, high morbidity, and high morbidity, not only in piglets but also in finishing pigs (Zhou et al., 2008; Zhou and Yang, 2010). The usage of HP-PRRSV-derived commercial killed and live vaccines has strengthened the control of PRRS on pig farms, but PRRS still occasionally outbreaks due to changes in the viral genome, virulence reversion of live vaccine strain, or the invasion of strain NADC30 (Han et al., 2009; Tian et al., 2009; Leng et al., 2012; Chen et al., 2015; Jiang et al., 2015).

Although the outbreak of African swine fever (ASF) in 2018 has concealed the problems caused by other diseases on pig farms, given the situation of PRRS in the United States and Demark, it is essential to know the real situation of PRRS epidemic in China, to understand the role of the virus' evolution, and to predict major problems in PRRS control (Kvisgaard et al., 2017; Zhao et al., 2019). Considering the complicated diversity of PRRSV, especially virus recombination, a sufficient number of the whole genome analysis of PRRSV might be beneficial to fully understand the real situation in the field. In this study, whole-genome analyses of 365 PRRSV isolates were used to evaluate the current status of PRRSV epidemiology, and moreover, further prospect of PRRSV evolution was predicted.

## Materials and Methods

### Sequence Samples

In total, 365 PRRSV sequences were gained from GenBank database, of which 353 strains were isolated in mainland China from 1996 to 2017 (Table S1).

### Sequence Alignment and Phylogenetic Analysis

The complete PRRSV genomic sequences were aligned with the online MAFFT software (http://mafft.cbrc.jp/alignment/server/) and ClustalW in the Lasergene software (DNASTAR Inc., Madison, version 7.1). Genetic recombination was analyzed with the Recombination Detection Program software (developed by the University of Cape Town, version 4.8). Positive genetic recombination was defined as more than four algorithm results that were positive and at least two *P*-values of the results were ≤ 10^−10^. Phylogenetic trees were constructed with the distance-based neighbor-joining method in the MEGA software (version 10.0), and the reliability of each tree was assessed with a bootstrap analysis using 1,000 replicates.

## Results

### Evolutionary Status of PRRSV in China

**Figure 1:**
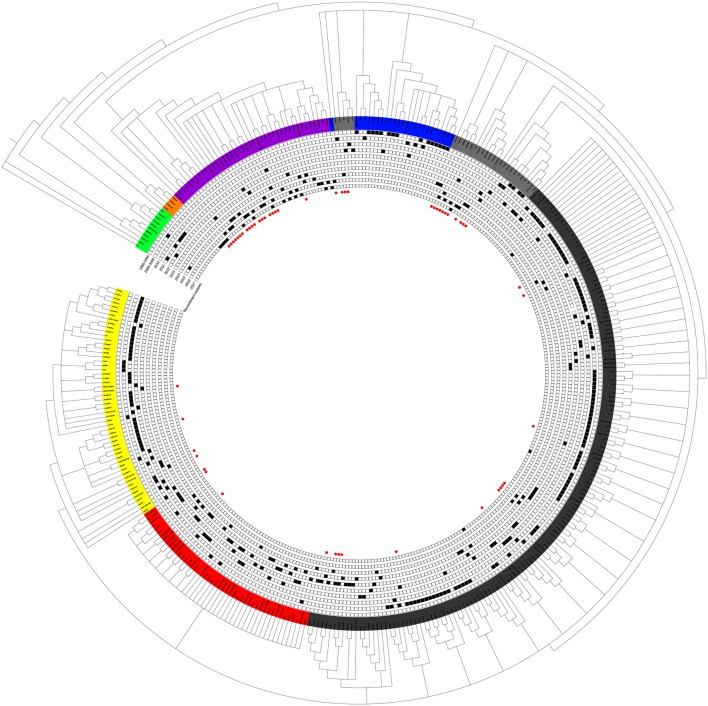
Phylogenetic tree based on the analysis of the complete genome sequences of PRRSV isolates. The total PRRSV isolates were divided into eight subgroups: EU-type strain, MN184-like strain, NADC30-like strain, Classic NA strain, Intermediate PRRSV strain, Original HP-PRRSV strain, JXA1-P80-like strain, and PRRSV2010 strain, which are marked in green, orange, purple, blue, gray, black, red, and yellow, respectively. The date of isolation of each individual strain is marked as a black solid square, and recombinant strains are shown with red dots in the inner cycle.

Although small sub-branches were detected, the total PRRSV isolates could be divided into eight subgroups: EU-type (EU), Classic North American (Classic NA), Original HP-PRRSV, PRRSV2010, JXA1-P80-like, MN184-like, NADC30-like, and Intermediate PRRSV (Figure 1).

**Figure 2:**
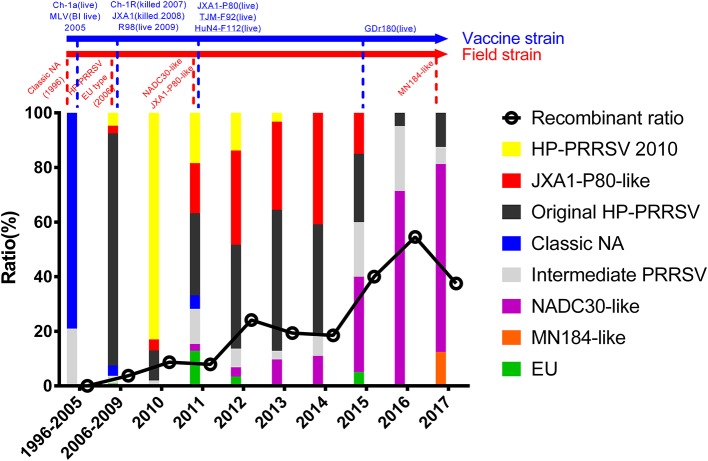
Tendency of PRRSV and trends in recombinant isolates in different periods. PRRSV isolates belonging to EU-type strain, MN184-like strain, NADC30-like strain, Classical PRRSV strain, Intermediate PRRSV strain, Original HP-PRRSV strain, JXA1-P80-like strain, and PRRSV2010 strain are showed in green, orange, purple, blue, gray, black, red, and yellow, respectively. Trends in recombinant isolates in different periods were shown. The representative field strains were shown in red, killed vaccine strains were shown in blue, and live vaccine strains were shown in blue with underline.

The epidemic of PRRSV isolates could be divided into four periods. In 1996–2005, most of the isolates belonged to the Classic NA group, together with several Intermediate PRRSV strains. In 2006–2010, with the outbreak of highly pathogenic PRRS, HP-PRRSV was the pandemic strain in pig farms in China. In 2010, most isolates were located on the same branch on the phylogenetic tree, which could be distinguishable from Original HP-PRRSV and was designated PRRSV2010. During the period 2011–2015, a new subgroup, designated JXA1-P80-like, diverged from Original HP-PRRSV, and the NADC30-like strain invaded China. Isolates belonging to seven subgroups were found in this period and, therefore, caused very genetically complex PRRSV epidemics. After 2015, the Original HP-PRRSV, NADC30-like, and Intermediate PRRSV strains were the main source of PRRSV isolates. In 2017, two isolates belonging to the MN184-like group were reported (Figures 1, 2).

### EU-Type, Classic NA, and PRRSV2010 Strains

EU-type isolates were found intermittently from 2006 to 2015 (Table S1 and Figure 1). These nine isolates had the same molecular marker as the LV strains and shared a high homology with strains NMEU09-1 and BJEU06-1 (data not shown).

**Figure 3:**
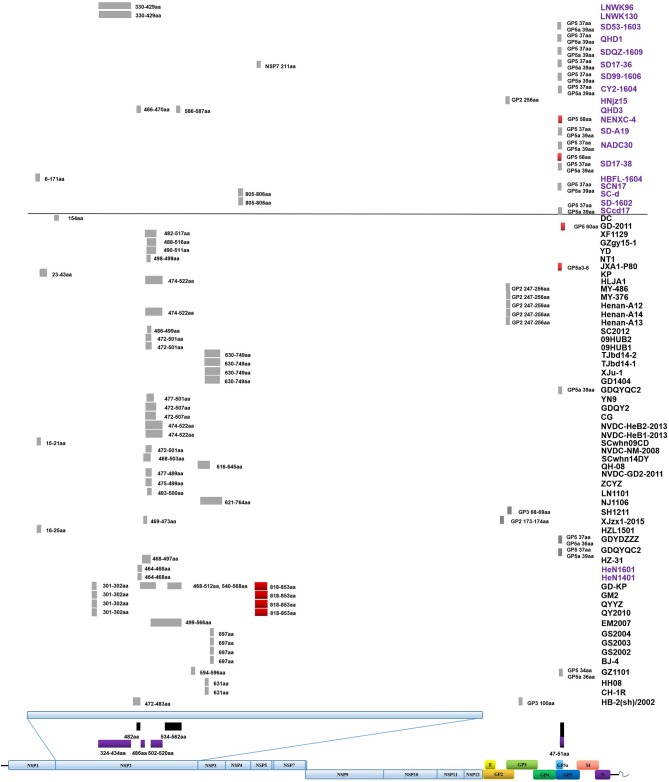
Amino acid deletions and insertions in PRRSV strains. The distinct deletions of HP-PRRSV and NADC30 are shown with black rectangles and purple rectangles, respectively. Deletions that are not consistent with HP-PRRSV or NADC30 are marked with gray rectangles. Insertions are shown with red rectangles. The rough positions of deletions and insertions are shown, and the precise positions are marked beside the rectangle. All sites of amino acid deletion or insertion are based on the sequence of strain VR2332. Strains with unique deletions relative to HP-PRRSV or NADC30 in the NSP2 region are marked with black or purple labels, respectively. Deletions and insertions of GX1001 are not shown in the figure.

The Classic NA strains had been the pandemic PRRSV strains in China, but disappeared rapidly after the HP-PRRS outbreak. The latest reports of Classic NA were in 2011, and deletions were found in several of these isolates (Figures 2, 3 and Table S1).

The first isolate in the PRRSV2010 subgroup was reported in 2008, and three isolates were reported in 2009, but these four strains could not be identified as a new subgroup. In 2010, a huge number of HP-PRRSV strains were reported and localized to this branch, after which several strains were reported in 2011, 2012, and 2013. However, there was no report of the PRRSV2010 strain after 2014 (Figures 1, 2 and Table S1). Two isolates (DC and XF1129) have an additional deletion in the NSP2-coding region and a small number of recombination events were detected in this subgroup. However, no unique genetic marker was detected in the PRRSV2010 subgroup (Figures 1, 3 and Table S1).

### JXA1-P80-Like Strains

Numbers of isolates from this subgroup were detected each year from 2011 to 2015, especially in 2012, 2013, and 2014, constituting nearly one-third of the total isolates in those years (Figure 2 and Table 1). Analysis of the 13 amino acids located in ORF1a, ORF1b, GP4, and GP5, which are unique to JXA1-P80 showed that 36 of the 41 isolates had ≥7 unique amino acids (Table 1; Jiang et al., 2015). Additional deletions in NSP2 which were not consistent with HP-PRRSV, were detected in some isolates, but no insertions were found in these strains, and only one isolate was recombinant strain (Figure 1 and Table S1).

**Table 1:** Analysis and comparisons of the unique amino acids in ORF1a, ORF1b, GP4, and GP5 among the JXA1-P80-like strains which isolated during 2011–2015.

**Year**	**Name**	**NO**.	**ORF1a**								**ORF1b**			**GP4**	**GP5**	**Total**
			**686 (D)**	**782 (T)**	**958 (V)**	**981 (R)**	**987 (G)**	**1,092 (M)**	**1,422 (L)**	**1,659 (D)**	**420 (G)**	**844 (H)**	**1,033 (T)**	**172 (V)**	**196 (L)**	**13**
2011	11NZ-GD	JX217036	**√**	**√**	**√**	**√**	**√**	**√**	**√**	**√**	**√**	**√**	**√**	**√**	**√**	13/13
	11XX-GD	JX235367	**√**	**√**	**√**	**√**	**√**	**√**	**√**	**√**	**√**	**√**	**√**	**√**	**√**	13/13
	11SH1-GD	JX235366	**√**	**√**	**√**	**√**	X	X	**√**	X	**√**	**√**	X	X	**√**	8/13
	NVDC-HeB1-2011	KP771749	**√**	**√**	**√**	**√**	**√**	X	**√**	**√**	**√**	**√**	X	X	**√**	10/13
	NVDC-JS2-2011	JQ715698	**√**	X	X	X	X	X	X	X	**√**	X	X	X	**√**	3/13
	11SH-GD	JX235365	**√**	**√**	**√**	**√**	X	X	**√**	X	**√**	**√**	X	**√**	**√**	9/13
	GX1001	JQ955657	X	X	X	X	X	X	X	X	**√**	**√**	**√**	X	**√**	4/13
2012	NVDC-BJ9-2012	KP771756	**√**	X	**√**	**√**	**√**	**√**	**√**	X	**√**	**√**	X	**√**	**√**	10/13
	NVDC-SD2-2012	KP771768	**√**	X	**√**	**√**	**√**	**√**	**√**	**√**	**√**	**√**	X	**√**	**√**	11/13
	NVDC-BJ3-2012	KP771762	**√**	**√**	**√**	**√**	**√**	X	X	**√**	**√**	**√**	X	X	**√**	9/13
	NVDC-BJ4-2012	KP771761	**√**	**√**	**√**	**√**	**√**	X	X	**√**	**√**	**√**	X	X	**√**	9/13
	NVDC-BJ5-2012	KP771760	**√**	**√**	**√**	**√**	**√**	X	X	**√**	**√**	**√**	X	X	**√**	9/13
	NVDC-SD1-2012	KP771769	**√**	X	**√**	**√**	**√**	**√**	X	**√**	**√**	**√**	X	**√**	**√**	10/13
	NT1	KP179402	**√**	**√**	**√**	**√**	X	**√**	**√**	**√**	**√**	**√**	**√**	**√**	**√**	12/13
	NT2	KP179403	**√**	**√**	**√**	**√**	**√**	**√**	**√**	**√**	**√**	**√**	X	X	**√**	10/13
	NT3	KP179404	**√**	**√**	**√**	**√**	**√**	X	**√**	**√**	**√**	**√**	**√**	**√**	**√**	11/13
	JL-04/12	JX177644	**√**	**√**	**√**	X	X	X	**√**	**√**	**√**	**√**	X	**√**	**√**	9/13
2013	HEB 20130008-13	KP771753	X	X	X	X	X	X	**√**	**√**	**√**	X	X	X	**√**	4/13
	NVDC-MD1-2013	KP771751	**√**	**√**	X	**√**	X	**√**	**√**	X	**√**	**√**	X	**√**	**√**	9/13
	NVDC-HBCZ-2013	KP771742	**√**	**√**	**√**	**√**	X	X	**√**	X	**√**	X	X	X	**√**	7/13
	NVDC-SDXX-2013	KP771741	**√**	X	**√**	**√**	**√**	X	**√**	**√**	X	**√**	**√**	X	**√**	9/13
	NVDC-MD2-2013	KP771750	**√**	**√**	**√**	**√**	X	**√**	X	X	**√**	**√**	X	**√**	**√**	9/13
	NVDC-BJPG-2013	KP771743	**√**	X	**√**	**√**	**√**	**√**	X	X	**√**	**√**	**√**	**√**	**√**	10/13
	NVDC-SXJC-2013	KP771740	**√**	X	**√**	**√**	X	X	X	X	**√**	**√**	**√**	**√**	**√**	8/13
	HNyc13	KT022072	**√**	**√**	**√**	**√**	**√**	**√**	**√**	X	**√**	**√**	**√**	**√**	**√**	12/13
	HEB-2013	KJ591659	**√**	**√**	**√**	**√**	X	X	**√**	**√**	**√**	**√**	X	X	**√**	9/13
	HEB 20130008-14	KP771752	**√**	**√**	**√**	**√**	**√**	X	**√**	**√**	**√**	**√**	X	X	**√**	10/13
2014–2015	NVDC-shh01-2014	KP771736	**√**	X	**√**	**√**	X	**√**	X	X	X	**√**	X	**√**	**√**	7/13
	HB-XL	KP162169	**√**	X	**√**	**√**	**√**	X	**√**	**√**	**√**	X	X	X	**√**	8/13
	NVDC-13SXJC-2014	KP771780	**√**	X	**√**	**√**	X	X	X	X	**√**	**√**	**√**	**√**	**√**	8/13
	NVDC-SHH02-2014	KP771735	**√**	**√**	**√**	**√**	X	**√**	**√**	X	X	**√**	X	X	**√**	8/13
	NVDC-SD6-2014	KP771737	**√**	X	**√**	**√**	X	X	X	**√**	X	**√**	X	X	**√**	6/13
	NVDC-SD1-2014	KP771738	**√**	**√**	**√**	**√**	**√**	X	**√**	**√**	**√**	**√**	**√**	**√**	**√**	10/13
	HNxa14	KT022071	**√**	X	**√**	**√**	**√**	**√**	**√**	X	**√**	**√**	**√**	**√**	**√**	11/13
	HENZK-1	KU950373	**√**	X	X	**√**	X	**√**	**√**	X	**√**	**√**	**√**	X	**√**	8/13
	HB2014001	KM261784	**√**	**√**	**√**	X	X	**√**	X	**√**	**√**	**√**	X	X	**√**	8/13
	HNP5	KT445876	**√**	X	**√**	**√**	**√**	X	X	**√**	**√**	**√**	X	X	**√**	8/13
	HUN-2014	KP330232	**√**	**√**	**√**	**√**	**√**	X	X	X	**√**	**√**	X	X	**√**	8/13
	HENPDS-2	KU950370	**√**	**√**	**√**	**√**	**√**	**√**	**√**	X	**√**	**√**	**√**	**√**	**√**	12/13
	GZgy15-1	KT358728	**√**	**√**	X	**√**	X	X	X	**√**	X	**√**	X	X	**√**	6/13
	JXja15	KR149645	**√**	X	**√**	**√**	X	X	**√**	**√**	**√**	**√**	**√**	X	**√**	9/13

*Amino acid which consistent with JXA1-P80 was shown as “**√**” with shadow background and isolates which share equal or more than 7 unique amino acids with JXA1-P80 were labeled with shadow background. PRRSV VR2332 was used as a reference for the position of amino acid sites*.

### NADC30-Like Strains

**Figure 4:**
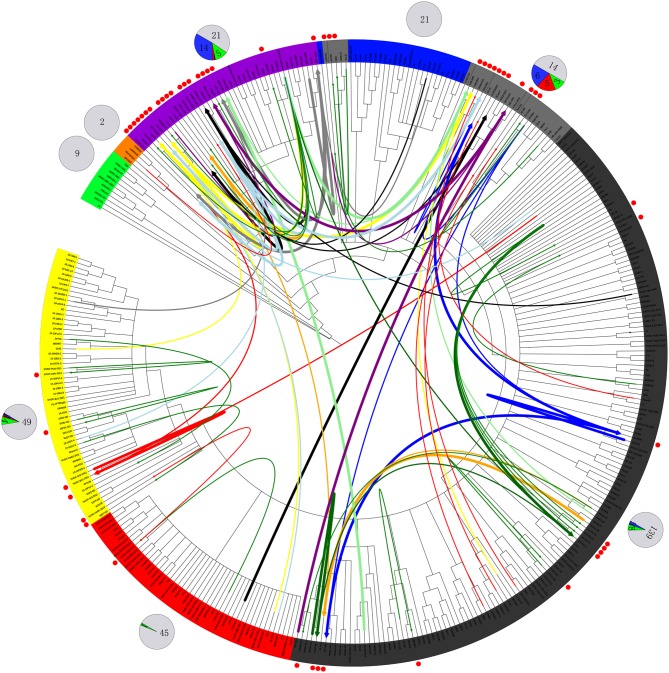
Recombination route and isolate ratio of PRRSV strains. EU-type strain, MN184-like strain, NADC30-like strain, Classic NA strain, Intermediate PRRSV strain, Original HP-PRRSV strain, JXA1-P80-like strain, and PRRSV2010 strain are marked with a background of green, orange, purple, blue, gray, black, red, and yellow, respectively. Recombinant strains are shown with red dots in the outer cycle. Recombinant strains belonging to the Inner group and Extensional group are shown with single thin green line and a thin red line, respectively. The major parent strains and minor parent strains of isolates belonging to the Propagating group are marked with the same color; major parent strains are shown with thick lines, and minor parent strains are shown with thin lines; lines 1 to 10 were marked with blue, red, light blue, black, yellow, orange, gray, light green, dark green and purple, respectively. The pie chart in the outer cycle represents the numbers of strains in the different recombinant and non-recombinant groups; the Inner group is shown in green, Extensional group is shown in red, Propagating group is shown in blue, and non-recombinant strains are shown in light gray. The number of strains in each group are labeled.

The first isolate, WUH6, was reported in 2011, and the strains in the NADC30-like subgroup increased rapidly from 2015 to 2017 (Figures 1, 2). However, seven isolates, 14LY01-FJ, 14LY02-FJ, 15LY01-FJ, 15LY02-FJ, SDlz1601, HeN1401, and HeN1601, which shared unique amino acid deletion with NADC30 were not located in the branch of the NADC30-like group. Instead they belonged to the Original HP-PRRSV and Intermediate NA groups, respectively (Figure 1 and Table S1). Deletions and insertions were found in these strains, and 20 of the 41 isolates are recombinant strains(Figures 3, 4).

### Intermediate PRRSV Strains

Strains belonging to the Intermediate PRRSV subgroup are complex and can be divided into several kinds of isolates. Strains GD3, NB/04, SHB, and HB-1(sh)/2002, which were isolated before 2006, do not have the unique 29-amino-acid deletion, which were used as a gene marker for HP-PRRSV (Table S1 and Figure 1). Strain NT0801, NJ1106, and HZ-31, which were located in the same small branch. NJ1106 and HZ-31 were recombinant strains, which were recombined by NT0801 with other strains (Table 2 and Figure 4). SDlz1601, HeN1401 SDhz1512, and HeN1601 are all recombinant isolates, arising from the recombination of NADC30 with other strains (Table 2 and Figure 4). The four strains QY2010, QYYZ, GM2, and GD-KP share a unique deletion (amino acids 301–302) and insertion (amino acids 813–848) in the NSP2-coding region and clustered on the same small branch of the phylogenetic tree (Figures 1, 3). Several isolates could not be classified (Figure 1). Recombination occurred frequently in this subgroup, and half the isolates were recombinant strains. Seven of the 10 strains that were isolated after 2015 were recombinant strains, and five of these seven recombinant strains had recombined from NADC30 (Figure 1 and Table 2).

**Table 2:** Information of recombine PRRSV isolates from 2006 to 2017.

	**Recombinant virus**	**Major parent**		**Minor parent**		**Breakpoint**		***P-val***						
		**Name-NO.-Year (similarity)**	**Region**	**Name-NO.-Year (similarity)**	**Region**	**Beginning(%)**	**Ending(%)**	**RDP**	**GENECONV**	**BootScan**	**MaxChi**	**Chimera**	**SiScan**	**3Seq**
Inner group	QH-08-KU201579-2008(HP-PRRSV)	FZ06A-MF370577-2006 (99.2%)(HP-PRRSV)	1-2449 3276-END	Ch-1R-EU807840-Attenuate (98.9%)	2450-3275	99	99	6.109*10-38	8.007*10-35	6.172*10-37	8.517*10-8	6.214*10-9	1.055*10-6	2.267*10-10
	09HUB7-GU168567-2009(HP-PRRSV)	HLJHL-HM189676-2009 (98.7%)(HP-PRRSV)	1-4447 7926-END	09HUB5-GU168568-2009 (99.7%)(HP-PRRSV)	4448-7925	99	99	6.907*10-14	5.013*10-13	1.125*10-7	4.997*10-6	9.077*10-9	1.746*10-10	1.301*10-12
	10BY-GD-JX192636-2010(PRRSV2010)	10-10GX-2-JQ663559-CHINA2010 (98%)(PRRSV2010)	1-4235 8836-END	YN9-GU232738-CHINA2008 (98.6%)(HP-PRRSV)	4236-8835	99	99	1.166*10-8	-	-	1.603*10-11	3.643*10-12	-	5.145*10-24
	10-10QN-JQ663556-2010(HP-PRRSV)	HPBEDV-EU236259-2007 (99.4%)(HP-PRRSV)	1-2686 4369-END	10-10GX-2-JQ663559-2010 (99.7%)(PRRSV2010)	2687-4368	99	99	1.922*10-15	1.213*10-13	2.846*10-14	1.946*10-6	8.248*10-7	1.149*10-6	1.133*10-10
	10-10FUJ-2-JQ663547-2010(PRRSV2010)	JN-HS-HM016158-2008 (99.6%)(PRRSV2010)	1-2513 4145-END	GX1002-JQ955658-2010 (99.4%) (JXA1-P80-like)	2514-4144	99	99	1.781*10-17	1.409*10-13	5.737*10-15	2.690*10-7	8.511*10-8	6.472*10-9	1.133*10-10
	10FS1-GD-JX192635-2010(PRRSV2010)	10HD-GD-JX215553 (99.6%)(PRRSV2010)	1-8037 9283-END	JXA1-P80-FJ548853 (99.9%)(JXA1-P80-like)	8038-9282	99	99	6.889*10-12	3.397*10-10	1.334*10-6	1.512*10-3	2.854*10-3	3.077*10-4	2.664*10-8
	GM2-JN662424-2011(IntermediatePRRSV)	QYYZ-JQ308798-2011 (99%)(Intermediate PRRSV)	1-7449 11027-END	RespPRRS_MLV-AF066183-Attenuate (99.4%) (attenuate strain)	7450-11026	99	99	2.607*10-220	1.181*10-215	-	2.903*10-42	1.163*10-42	5.545*10-49	9.066*10-10
	NJ-1106-JX880029-2011(IntermediatePRRSV)	NT0801-HQ315836-2008(99.8%) (Intermediate PRRSV)	1-223 3692-END	YD-JF748717-2009 (100%)(JXA1-P80-like)	224-3691	99	99	7.108*10-50	4.918*10-48	1.125*10-37	7.411*10-24	1.308*10-24	2.579*10-26	1.813*10-10
	11GZ-GD-JX235370-2011(HP-PRRSV)	11FS11-GD-JX215551-2011 (100%)(HP-PRRSV)	223-12061	NVDC-CQ1-2011-KP771746-2011 (99.4%)(HP-PRRSV)	1-222 12062-END	99	99	3.687*10-34	3.480*10-27	3.218*10-18	1.645*10-17	4.393*10-15	4.605*10-12	2.323*10-57
	NVDC-HeN-2012-KP771771-2012(PRRSV2010)	NVDC-HuN-2011-KP771770-2012 (98.6%)(PRRSV2010)	1-6588 7929-END	NVDC-BJ5-2012-KP771760-2012 (99.9%)(JXA1-P80-like)	6589-7928	99	99	6.146*10-24	4.385*10-22	4.504*10-20	1.054*10-5	1.317*10–5	3.128*10-8	4.061*10-10
	NVDC-SD2-2012-KP771768-2012(JXA1-P80-like)	JXA1-P80-FJ548853-Attenuate (99.7%)(JXA1-P80-like)	1-4107 5091-END	Unknown (NVDC-HeB2-2012-KP771772-2012)(HP-PRRSV)	4108-5090	99	99	3.413*10-13	9.311*10-12	-	2.266*10-2	2.217*10-2	3.692*10-8	1.624*10-8
	HeNan-A9-KJ546412-2013(HP-PRRSV)	Henan-A5-KJ534540-2013 (96%)(HP-PRRSV)	1366-11405	Unknown(Henan-A8-KJ534543-2013)(HP-PRRSV)	1-1365 11406-END	99	99	3.859*10-14	3.442*10-19	9.088*10-20	2.545*10-3	1.696*10-5	2.119*10-21	1.050*10-4
	FJL15-KY412887-2014(NADC30-like)	NADC30-JN654459-2008 (95.1%)(NADC30-like)	1-4613 5785-END	GD1404-MF669720-2014 (99.3%)(HP-PRRSV)	4614-5784	99	99	1.992*10-124	2.437*10-124	-	3.462*10-26	7.228*10-26	1.705*10-28	3.294*10-9
	HENXX-1-KU950372-2014(NADC30-like)	NADC30-JN654459-2008 (96.3%)(NADC30-like)	1-13944 14223-END	KP-GU232735-2008 (94.9%)(HP-PRRSV)	13945–14222	99	99	1.550*10–21	1.107*10–6	7.769*10–20	7.733*10–3	3.806*10–4	4.597*10–5	3.294*10–9
	14LY01-FJ-KP780881-2014(HP-PRRSV)	FZ06A-MF370557-2006 (99.2%)(HP-PRRSV)	1-1624 3635-END	NADC30-JN654459-2008 (93.6%)(NADC30-like)	1625–3634	99	99	5.479*10–173	1.209*10–135	4.581*10–80	1.496*10–41	4.020*10–40	9.804*10–35	3.294*10–9
	14LY02-FJ-KP780882-2014(HP-PRRSV)	FZ06A-MF370557-2006 (97.1%)(HP-PRRSV)	1-1625 3628-END	NADC30-JN654459-2008 (93.6%)(NADC30-like)	1626–3627	99	99	6.986*10–167	4.137*10–128	2.474*10–91	1.141*10–39	1.072*10–38	1.955*10–37	4.361*10–10
	HENZMD-9-KU950374-2015(NADC30-like)	NADC30-JN654459-2008 (95.5%)(NADC30-like)	1419-15425	HEB 20130008-13-KP771753-2013 (97.5%)(JXA1-P80-like)	1-1418 15426-END	99	99	4.609*10–106	2.512*10-81	9.363*10-104	2.587*10-23	2.284*10-25	1.190*10-38	4.000*10-9
	HNyc15-KT945018-2015(NADC30-like)	NADC30-JN654459-2008 (95.4%)(NADC30-like)	1-12215 14445-END	XJzx1-2015-KX689233-2015 (91%)(Intermediate PRRSV)	12216-14444	99	99	1.780*10-71	-	1.518*10-69	2.829*10-21	4.425*10-3	1.919*10-29	4.800*10-9
	GD-KP-KU978619-2015(Intermediate PRRSV)	QYYZ-JQ308798-2011 (95.7%)(Intermediate PRRSV)	1-699 2107-END	GD-P100-Attenuate (95.9%)	700-2106	99	99	8.691*10-53	2.210*10-36	4.915*10-43	4.447*10-14	2.165*10-3	6.733*10-35	2.400*10-9
	15LY02-FJ-KU215417-2015 (HP-PRRSV)	FZ06A-MF370557-2006 (98.3%) (HP-PRRSV)	1-1704 3628-END	NADC30-JN654459-2008 (92.8%)(NADC30-like)	1705-3627	99	99	6.986*10-167	4.137*10-128	2.474*10-91	1.141*10-39	1.072*10-38	1.955*10-37	4.361*10-10
	CY1-1604-MH651736-2016(NADC30-like)	NADC30-JN654459-2008 (95%)(NADC30-like)	1-3539 7383-END	10FS-GD-JX192634-2010 (97.4%)(HP-PRRSV)	3540-7382	99	99	3.335*10-207	1.587*10-189	6.519*10-197	1.016*10-46	8.509*10-49	8.180*10-48	1.023*10-9
	HBFL-1604-MH651739-2016(NADC30-like)	NADC30-JN654459-2008 (95.1%)(NADC30-like)	1-5721 6883-END	GD-EU825724-2007 (95%)(HP-PRRSV)	5722-6882	99	99	2.034*10-58	6.521*10-29	7.206*10-58	8.065*10-15	1.434*10-15	4.696*10-23	6.140*10-9
	Em2007-EU262603-2007(IntermediatePRRSV)	Unknown (SHB-EU864232-CHINA2005)(Intermediate PRRSV)	1-3299 4722-END	WUH1-EU187484-2006 (99.7%)(HP-PRRSV)	3300-4721	99	99	1.415*10-17	2.094*10-22	2.054*10-9	8.721*10-7	1.343*10-7	3.031*10-10	2.267*10-10
Extensional group	SH1211-KF678434-2012(IntermediatePRRSV)	07NM-FJ393456-2007 (95.7%)(HP-PRRSV)	1-13709 14399-END	QYYZ-JQ308798-2011 (93.2%)(Intermediate PRRSV)	13710-14398	99	99	1.622*10-32	-	-	9.039*10-8	1.051*10-10	1.518*10-6	2.030*10-9
	NVDC-HeB1-2012-KP771773-2012(PRRSV2010)	Unknown (NVDC-SD2-2012-KP771768-2012)(JXA1-P80-like)	1-3988 4939-END	NVDC-BJ3-2012-KP771762-2012 (99.7%) (JXA1-P80-like)	3989-4938	99	99	7.525*10-14	2.085*10-10	1.252*10-8	1.081*10-2	7.373*10-4	2.732*10-7	1.624*10-9
	HLJB1-KT351740-2013(IntermediatePRRSV)	Unknown (HeN301-MF766470-2013)(HP-PRRSV)	1-883 3017-END	HuN4-F112-Attenuate (99.9%)	884-3016	99	99	6.346*10-29	3.947*10-32	1.879*10-30	1.756*10-10	1.289*10-10	2.723*10-18	7.010*10-11
	SDhz1512-KX980392-2015(IntermediatePRRSV)	JXwn06-EF641008-2006 (94.7%)(HP-PRRSV)	1-11711 15465-END	NADC30-JN654459-2008 (95.5%)(NADC30-like)	11712-15464	99	99	2.005*10-103	1.935*10-61	7.722*10-102	4.529*10-34	8.558*10-11	2.409*10-38	3.200*10-9
	HNJYH-1606-MH651740-2016(NADC30-like)	Minnesota1-KP283414-2012 (90.9%)(MN184-like)	1-10934 12109-END	GD-EU825724-2007 (98.6%)(HP-PRRSV)	10935-12108	99	99	1.762*10-71	2.334*10-75	1.303*10-71	7.291*10-16	2.220*10-14	1.853*10-39	5.301*10-7
	GDZS2016-MH046843-2016(IntermediatePRRSV)	GD-EU825724-2007 (97.1%) (HP-PRRSV)	1-12673 14911-END	XJzx1-2015-KX689233-2015 (92.5%)(Intermediate PRRSV)	12674-14910	99	99	3.959*10-56	-	5.694*10-52	3.304*10-24	2.832*10-22	1.538*10-9	3.070*10-9
Propagating group Line 1	SY0909-HU315837-2009(HP-PRRSV)	AH0701-GU461292-2007 (99.3%)(HP-PRRSV)	1-11331 14165-END	NT0801-HQ315836-2008 (99.6%)(Intermediate PRRSV)	11332-14164	99	99	4.462*10-30	5.037*10-26	1.103*10-27	1.071*10-7	7.145*10-10	2.176*10-10	1.133*10-10
	HZ-31-KC445138-2012(IntermediatePRRSV)	SY0909-HQ315837-2009 (97.1%)(HP-PRRSV)	1-11641 15151-END	Unknown (NT0801-HQ315836-2008)(Intermediate PRRSV)	11642-15150	99	99	6.191*10-48	5.641*10-54	1.827*10-52	1.125*10-21	8.426*10-25	6.220*10-50	4.061*10-10
	SDlz1601-KX980393-2016(IntermediatePRRSV)	HENAN-HEB-KJ143621-2012 (91.3%)(NADC30-like)	1-3543 8162-END	HZ-31-KC445138-2012 (95.7%)(Intermediate PRRSV)	3878-8654	99	99	4.068*10-47	3.648*10-55	3.349*10-70	7.828*10-32	6.003*10-30	1.032*10-36	2.894*10-9
	MY-486-KJ609516-2013(HP-PRRSV)	SY0909-HQ315837-2009 (98.5) (HP-PRRSV)	1-11623 14535-END	Unknown (NT0801-HQ315836-2008)(Intermediate PRRSV)	11624-14534	99	99	1.609*10-53	6.349*10-54	3.225*10-59	5.563*10-22	1.235*10-21	1.937*10-33	4.061*10-10
Line 2	NVDC-BJ1-2012-KP771764-2012(PRRSV2010)	NVDC-BJ2-2012-KP771763-2012 (99.8%)(PRRSV2010)	1-4226 7870-END	WUH4-JQ326271-2011 (99.7%) (HP-PRRSV)	4227-7869	99	99	1.092*10-26	1.475*10-22	-	2.854*10-12	1.302*10-11	1.476*10-11	4.061*10-10
	SCcd17-MG914067-2017(NADC30-like)	SD53-1603-MH651744-2016 (94.8%)(NADC30-like)	1-5463 9821-END	NVDC-BJ1-2012-KP771764-2012 (96.2%)(PRRSV2010)	5464-9820	99	99	2.403*10-117	7.070*10-118	1.194*10-114	9.321*10-32	9.268*10-31	9.153*10-45	3.579*10-9
Line 3	HENAN-HEB-KJ143621-2012(NADC30-like)	NADC30-JN654459-2008 (96.6%)(NADC30-like)	1-1270 2043-END	10-10GX-4-JQ663561-2010 (96.8%)(PRRSV2010)	1271-2042	99	99	2.475*10-109	1.070*10-90	1.128*10-47	1.101*10-20	1.339*10-21	2.363*10-25	2.437*10-9
	SDYG1606-KY053458-2016(NADC30-like)	HENAN-HEB-KJ143621-2012 (92.9%)(NADC30-like)	1-5531 8869-END	JL-0412-JX177644-2012 (97.9%)(JXA1-P80-like)	5532-8868	99	99	2.223*10-127	1.166*10-125	2.395*10-123	6.591*10-40	1.193*10-12	8.468*10-43	3.070*10-9
	FJLIUY-2017-MG011718-2017(NADC30-like)	HENAN-HEB-KJ143621-2012 (90.9%)(NADC30-like)	1-6966 9537-END	GD2007-EU880433-2007 (94.3%)(HP-PRRSV)	6967-9536	99	99	1.377*10-53	2.791*10-22	-	8.162*10-24	4.231*10-27	9.498*10-48	3.579*10-9
	SDlz1601-KX980393-2016(IntermediatePRRSV)	HENAN-HEB-KJ143621-2012 (91.3%)(NADC30-like)	1-3543 8162-END	HZ-31-KC445138-2012 (95.7%)(HP-PRRSV)	3878-8654	99	99	4.068*10-47	3.648*10-55	3.349*10-70	7.828*10-32	6.003*10-30	1.032*10-36	2.894*10-9
Line 4	HENAN-XINX-KF611905-2013(NADC30-like)	NADC30-JN654459-2008 (96.6%)(NADC30-like)	1-4741 6485-END	NVDC-NM-2008-KP7717792008 (90.5%)(HP-PRRSV)	4742-6484	99	99	6.125*10-55	-	-	6.162*10-17	2.879*10-17	3.817*10-24	2.437*10-9
	SCN17-MH078490-2017(NADC30-like)	HENAN-XINX-KF611905-2013 (92.2%)(NADC30-like)	1-8535 11280-END	Clone20-FJ899592-2003 (98.9%)(Classic PRRSV)	8536-11279	99	99	1.975*10-85	9.501*10-80	1.337*10-83	5.014*10-24	1.505*10-24	2.243*10-27	4.772*10-9
	SCya17-MH324400-2017(IntermediatePRRSV)	11SH-GD-JX235365-2011 (93.3%)(JXA1-P80-like)	1-11683 12568-END	SCN17-MH078490-2017 (99.5%)(NADC30-like)	11684-12567	99	99	3.147*10-71	4.892*10-74	8.387*10-66	9.407*10-17	2.688*10-9	1.180*10-30	4.772*10-9
Line 5	JL580-KR706343-2013(NADC30-like)	NADC30-JN654459-2008 (94.2%)(NADC30-like)	1-5345 7366-END	09JS-JF268675-2009 (97.5%)(HP-PRRSV)	5346-7365	99	99	2.329*10-85	1.344*10-76	-	3.308*10-29	4.584*10-31	1.656*10-30	1.624*10-9
	SD-1602-MH651743-2016(NADC30-like)	JL580-KR706343-2013 (90.6%)(NADC30-like)	1943-15463	NT1-KP179402-2012 (97.8)(JXA1-P80-like)	1-1942 15464-END	99	99	1.454*10-59	1.296*10-101	4.350*10-95	6.492*10-20	3.413*10-28	2.563*10-45	1.023*10-9
	HeN1601-MF766474-2016 (IntermediatePRRSV)	SD-1602-MH651743-2016 (93%) (NADC30-like)	1-4076 12238-END	GD1404-MF669720-2014 (99.4%) (HP-PRRSV)	4077-12237	99	99	5.767*10-139	1.727*10-138	8.154*10-121	6.041*10-48	6.8.41*10-19	6.337*10-81	1.772*10-9
Line 6	HLJA1-KT351739-2013(HP-PRRSV)	Henan-A8-KJ534543-2013 (97.9%)(HP-PRRSV)	957-11505	Unknown (Henan-A5-KJ534540-2013)(HP-PRRSV)	1-956 11506-END	99	99	4.228*10-18	8.585*10-19	-	1.344*10-10	6.823*10-3	7.811*10-15	4.061*10-10
	SDQD-1604-MH651742-2016(NADC30-like)	NADC30-JN654459-2008 (94.8%)(NADC30-like)	1-6731 8143-END	HLJA1-KT351739-2013 (97.6%)(HP-PRRSV)	6732-8142	99	99	1.897*10-101	6.307*10-86	1.676*10-95	1.185*10-18	1.605*10-20	9.485*10-23	5.116*10-9
	SD53-1603-MH651744-2016(NADC30-like)	SDQZ-1609-MH651746-2016 (96.3%)(NADC30-like)	1-12725 13213-END	SD1-100-GQ914997-2009 (95.5%)(Classic PRRSV)	12726-13212	99	99	4.225*10-41	4.568*10-35	5.106*10-35	7.638*10-8	1.578*10-9	3.761*10-17	6.140*10-9
	SCcd17-MG914067-2017(NADC30-like)	SD53-1603-MH651744-2016 (94.8%)(NADC30-like)	1-5463 9821-END	NVDC-BJ1-2012-KP771764-2012 (96.2%)(PRRSV2010)	5464-9820	99	99	2.403*10-117	7.070*10-118	1.194*10-114	9.321*10-32	9.268*10-31	9.153*10-45	3.579*10-9
	SD17-38-MH068878-2017(NADC30-like)	SD53-1603-MH651744-2016 (95.2%)(NADC30-like)	1-6807 7423-END	10-10FUJ-1-JQ663546-2010 (96.6%)(PRRSV2010)	6808-7422	99	99	4.757*10-50	2.083*10-38	-	2.359*10-9	5.179*10-11	1.576*10-22	7.158*10-9
Line 7	HeN1401-MF766471-2014(Intermediate PRRSV)	NADC30-JN654459-2008 (95.4%)(NADC30-like)	1907-15449	Henan-A6-KJ534541-2013 (98%)(HP-PRRSV)	1-1906 15450-END	99	99	1.991*10-109	2.106*10-104	1.585*10-103	1.264*10-33	1.384*10-34	9.274*10-61	1.317*10-9
	SDZC-1609-MH651747-2016(NADC30-like)	Unknown (HeN1401-MF766471-2014)(Intermediate PRRSV)	1-13879 15463-END	HuN4-F112-Attenuate (99.9%)	13880-15462	99	99	5.016*10-150	1.832*10-147	1.367*10-139	2.093*10-26	2.470*10-26	1.057*10-29	2.046*10-9
Line 8	15LY01-FJ-KU215416-2015(HP-PRRSV)	FZ06A-MF370557-2006 (97.4%)(HP-PRRSV)	1-1625 3628-END	NADC30-JN654459-2008 (93.6%)(NADC30-like)	1626-3627	99	99	6.986*10-167	4.137*10-128	2.474*10-91	1.141*10-39	1.072*10-38	1.955*10-37	4.361*10-10
	HeN1501-MF766472-2015(HP-PRRSV)	HeNan-A2-KJ002452-2013 (99.5%)(HP-PRRSV)	1-12357 13329-END	Unknown (15LY01-FJ-KU215416-2015)(Intermediate PRRSV)	12358-13328	99	99	3.384*10-39	2.237*10-31	1.141*10-37	4.778*10-11	3.645*10-11	2.297*10-22	8.001*10-10
Line 9	HZL1501-MF669721-2015(IntermediatePRRSV)	MY-376-KJ609517-2013 (91.9%)(HP-PRRSV)	1-14189 15465-END	Unknown (Ingelvac ATP-DQ988080-Atteunate)(Classic PRRSV)	14190-15464	99	99	1.446*10-10	-	-	5.832*10-4	6.611*10-5	5.387*10-11	5.964*10-6
	HNJYF-1606-MH651738-2016(NADC30-like)	SC-d-MF375261-2015 (93.6%)(NADC30-like)	1-12137 12971-END	HZL1501-MF669721-2015 (93.3%)(Intermediate PRRSV)	12138-12970	99	99	7.595*10-26	-	1.332*10-22	1.190*10-4	3.925*10-6	2.894*10-5	4.093*10-9
Line 10														

*1,2 and 3 in cycle represent the order of isolates*.

### Deletions and Insertions

Deletions mainly occur in the NSP2-coding region, although several deletions were found in ORF2, ORF3, ORF5a, ORF5, and ORF1b (Table S1 and Figure 3). In addition to the amino acid 1 + 29 deletion, most of the HP-PRRSV strains (Original HP-PRRSV, PRRSV2010, and JXA1-P80-like) show the deletion of amino acids 47–51 in GP5a, consistent with the NADC30 and NADC30-like strains (Table S1). Several strains share the same deletions, including amino acids 474–522 (NVDC-HeB1-2013, NVDC-HeB2-2013, HLJA1, and Henan-A14), amino acids 464–468 (HeN1401 and HeN1601), amino acids 472–501 (NVDC-NM-2008, 09HUB1, and 09HUB2), amino acids 472–507 (CG and GDQY2), amino acids 805–806 (SD-1602 and SC-d), amino acid 697 (BJ-4, GS2002, GS2003, and GS2004), and amino acid 631 (CH-1R and HB-2(sh)/2002) in NSP2; amino acids 247–256 (Henan-A12, Henan-A13, Henan-A14, and MY-486) in GP2; and amino acid 37 in GP5 and amino acid 39 in GP5a (GDYDZZZ, GDQYQC2, SCN17, SCcd17, SD17-38, NADC30, SD-A19, CY2-1604, SD99-1606, SD17-36, SDQZ-1609, QHD1, and SD53-1603) (Table S1 and Figure 3). The LNWK96 and LNWK130 strains do not have the same deletions as MN184, although they belong to the MN184-like subgroup (Table S1 and Figure 3).

Insertions are only found in the NSP2-coding region and ORF5 (Figure 3). The insertion at amino acids 818–853 in the NSP2 region can be considered a marker of strains QY2010, QYYZ, GM2, and GD-KP, and insertions at amino acids 58 and 60 in the ORF5 coding region were detected in several isolates (Table S1 and Figure 3). However, the deletions and insertions in GX1001 are not discussed here because the deletion and insertion positions are specific (Table S1).

### Recombination

**Figure 5:**
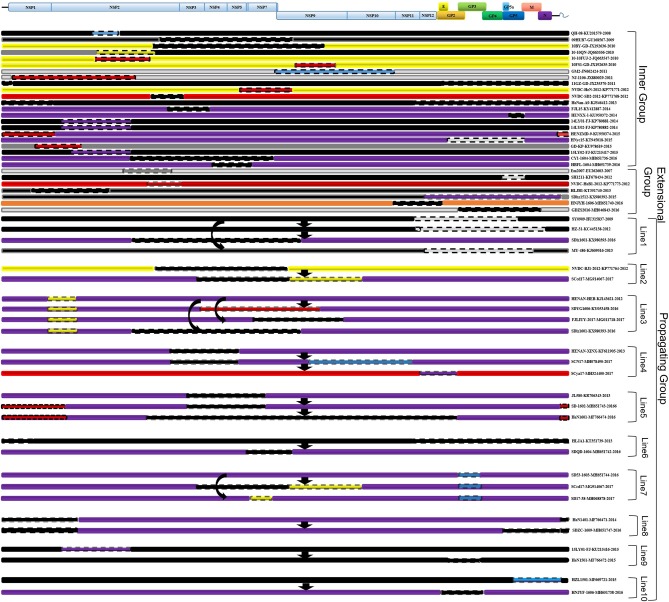
Recombinant region in each isolate. EU-type strain, MN184-like strain, NADC30-like strain, Classic NA strain, Intermediate PRRSV strain, Original HP-PRRSV strain, JXA1-P80-like strain, and PRRSV2010 strain are marked with green, orange, purple, blue, gray, black, red, and yellow rectangles, respectively. Major parental strains are shown with solid lines and minor parental strains with dotted lines. The rough sites of recombination are based on strain VR2332.

No recombinant strain was detected before 2006. In 2006–2011, the recombination rate was <10%. In 2012–2014, the recombination rate increased and remained at 20–30%. After 2015, the rate of recombination increased dramatically, to >35% (Figure 2). No recombinant strain was found in the EU, MN184-like, or Classic NA subgroup, but recombinant strains occurred frequently in the NADC30-like (20 of 41) and Intermediate PRRSV subgroups (14 of 28 strains). The recombination rate among strains isolated after 2015 in these two subgroups were 16/33 and 7/10, respectively (Figures 1, 4). Low recombination rates were detected in the three HP-PRRSV subgroups (Original HP-PRRSV, PRRSV2010, and JXA1-P80-like), but they were the parental strains in many recombination events (Table 2 and Figure 4). Unlike deletions, recombination events were found in almost every part of the PRRSV genome, rather than in the most genetically diverse regions (NSP2 and ORF5) (Table 2 and Figure 5). The recombination events could be divided into three types: (1) in which the Inner group was the major parental strain and the recombinant strain was located in the same subgroup; (2) in which the Extensional group was the major parental strain and the recombinant strains were located in different subgroups; and (Akay et al., 2004) in which the Propagating group's recombinant strain had generations (Table 2). Location of the recombinant strains in the phylogenic tree was changed in the Extensional group and Propagating group (part of the isolates) (Figure 4 and Table 2). Ten isolates that produced progeny viruses by recombination were identified, and the complete routes, from the first strain to the last progeny strain, were traced and designated route 1 to route 10 (line 1 to 10, respectively) (Table 2). Among these 10 lineages, propagation during recombination could be classified into three types. In type 1, the recombination events occurred in different parts of the genome, generating multiple recombinant strains (lineage 4 from HENAN-XINX to SCN17, lineages 2, 5, 7, and 8). In type 2, the recombination events occur in a similar position, but the length of recombination position varies (lineage 1 from SY0909 to HZ-31, from SY0909 to MY-486; and lineage 3). In type 3, the recombination site changed completely after propagation (lineage 1 from HZ-31 to SDlz-1601; lineage 4 from SCN17 to SCya17; lineages 6, 9, and 10) (Table 2 and Figure 5).

## Discussion

PRRS outbreaks were considered as the most pandemic and destructive disease in swine herds in mainland China. Variant PRRSV strains isolated from pig farms have different genetic characteristics, including insertions, deletions, and recombination. Vaccine-like strains, invasive strains, and recombinant strains have been reported, making the current epidemic status of PRRSV both complex and confusing (Yan et al., 2013; Chen et al., 2015; Jiang et al., 2015; Lu et al., 2015; Wang et al., 2015; Zhao et al., 2015; Zhang et al., 2016). To fully understand the current evolutionary status of PRRSV and explain the possible reasons for that status, 353 strains that were isolated from mainland China were analyzed. The total PRRSV strains could be divided into eight subgroups, but no isolate belonging to the EU, Classic NA, PRRSV2010, or JXA1-P80-like subgroup has been reported since 2016 (Figures 1, 2), indicating that strains belonging to these four subgroups are not the main source of the currently circulating PRRSV. For Classic NA subgroup, from our results, two strains GM2 and QH-08 were recombinant strains, which were recombined by vaccine strain (Classic MLV). GM2 was recombined by RespPRRS MLV, but we found several isolates including QY2010, QYYZ, and GD-KP, which had the same genome characteristics and co-located in the small branch and were all isolated from Guangdong province. Strain QH-08 was also a recombinant strain, which has one parent strain that is attenuate vaccine strain Ch-1R. These results indicated that vaccine strain may take, in part, in the recombination of PRRSV and influence PRRSV revolution. However, considering the awareness of rational usage of live vaccine in pig farms and the less competitiveness of Classic NA strain in replication and infection with HP-PRRSV strain, isolates that belonged to this subgroup will neither disappear nor play a major role in PRRSV outbreak, and referring to previous studies, a limited epidemic might happen either by recombination or by vaccine use in the future (Li et al., 2009). The most recent report of the EU subgroup was in 2015, but no further information on it could be found. The EU isolates had higher homology with strain LV than Lena, for the same reasons of pandemic type 1 EU strain as Classic NA strain, and besides the study published before, there was a high suspicion of the usage of commercial EU type vaccine in the EU isolates in China (Wang X. et al., 2016). Also, because of the awareness of rational usage of vaccine, the EU type could be rarely found in the future. MN184-like strains were reported in 2017 in Liaoning province, but in 2018, the outbreak of ASF first happened in Liaoning province. The short transmission time and control method of ASF were not fit for virus transmission, and the same situation would have happened in Fujian province (Liu et al., 2019) (http://www.moa.gov.cn/nybgb/2017/dsq/201802/t20180201_6136188.htm). Isolates of the PRRSV2010 subgroup disappeared rapidly after 2011, and no specific amino acid site changes were detected in this subgroup. It might have occurred by an accidental event as described before (Yu et al., 2012; Shi et al., 2013).

The most important events of PRRSV epidemics in the past 10 years were the virulence reversion of JXA1-P80 and the invasion of strain NADC30, both of which occurred in 2011, and unfortunately, neither of these events was recognized immediately. The virulence reversion of JXA1-P80 was first reported by our group and, subsequently, by many other groups (Jiang et al., 2015; Zhao et al., 2017). In those studies, most of the isolates had the same unique amino acids as JXA1-P80, suggesting that they arose from virulence reversion of the vaccine strain. Recent reports have shown that JXA1-P80-like strains have spread to almost all the major pig-farming provinces in China and can recombine with other strains (Dong et al., 2017; Liu et al., 2017; Zhao et al., 2017; Wang et al., 2018; Guo et al., 2019). All these findings suggest that the JXA1-P80-like viruses should be responsible for the PRRS outbreaks in the past few years.

The first NADC30-like strain, WUH6, was isolated in 2011, but it was reported to be a novel strain with longer deletion in NSP2 region rather than as an invasive NADC30 strain. It seems that NADC30-like strains have a strong ability to recombine with other strains. The increasing numbers of NADC30-like isolates and the increasing recombination rate indicate that the recombination of NADC30-like strains has played an important role in PRRS outbreaks (Figure 2). This inference has been supported by recent reports (Sui et al., 2018; Zhou et al., 2018c; Guo et al., 2019; Zhang H. et al., 2019).

The increasing numbers of strains from the Intermediate PRRSV subgroup may indicate a trend in viral evolution, especially those isolates that arose from recombination among the NADC30-like, Original HP-PRRSV, JXA1-P80-like, PRRSV2010, and Classic NA subgroups after 2015 (Figures 4, 5 and Table 2). This tendency showed that the evolution of PRRSV turned from traditional subgroups to interval place, which happened before HP-PRRSV that occurred in 2006 (An et al., 2010). At that time, deletions played the major role in the evolution of PRRSV, whereas recombination now does so. This increase in recombinant strains may be attributable to the blending of different strains in the same area. Newly recombinant strains may gain characteristic that adapt them to their circumstances, and strains that have integrated the advantages of their parental strains present a great challenge to PRRS control.

Viral evolution is driven by mutation, deletion, insertion, and recombination, and several studies had demonstrated the effects of amino acid mutations on virulence (Zhou et al., 2009; Zhao et al., 2018). Compared with insertion, other changes were found more frequently in the PRRSV genome; unlike mutation and recombination, which might happen in all PRRSV genome, deletions were mainly found in the NSP2 and ORF5 regions and often mentioned as characteristics of a[vanji] new strain.

During the highly pathogenic PRRS outbreak, the 1 + 29 amino acid deletion was used as a specific gene marker to identify HP-PRRSV, although it did not influence the virulence (Zhou et al., 2009). Compared with the ORF5a coding region of HP-PRRSV with Classic NA, an early ending code was found at the end of ORF5a. This caused the additional 5aa deletions in the GP5a of HP-PRRSV. This result was supported by a recent report. The influence of the 5aa deletion on virulence and viability need further investigation (Zhang et al., 2017). Deletion could influence PRRSV virulence and was supported by strains NT2 and XJu-1, which were both isolated in 2012 (Table S1). NT2 is highly pathogenic, and XJu-1 is moderately virulent; the major genetic difference between the two strains is that XJu-1 has an additional deletion at position 630–749aa of NSP2 (Table S1) (Jiang et al., 2015; Xia et al., 2015). The deletion of 37aa in GP5 and 39aa in GP5a has been detected in many strains (GDYDZZZ, GDQYQC2, SCN17, SD17-38, NADC30, SD-A19, CY2-1604, SD99-1606, SD17-36, SDQZ-1609, QHD1, and SD53-1603), although these strains were isolated in different provinces at great distances and belong to different subgroups (Figure 3). Among these strains, SCN17, SD17-38, NADC30, SD-A19, CY2-1604, SD99-1606, SD17-36, SDQZ-1609, QHD1, and SD53-1603 were NADC30-like strains, but GDYDZZZ and GDQYQC2 belonged to the Intermediate PRRSV subgroup, and there is no evidence that showed that strain GDYDZZZ or GDQYQC2 was active in a recombination event. This result suggests that the same deletions can occur independently in different places. Besides, the 37aa deletion in GP5 was in the neutralizing epitope, and it might cause immunogenicity change (Ostrowski et al., 2002). Similar situations were also found in other deletions, for example, the deletion of amino acids 474–522 in NSP2 has been detected in several strains (NVDC-HeB1-2013, NVDC-HeB2-2013, HLJA1, and Henan-A14) that were isolated in Hebei, Henan, and Heilongjiang Provinces; the deletion of amino acids 472–501 in NSP2 has been detected in NVDC-NM-2008, 09HUB1, and 09HUB2, which were isolated in Inner Mongolia and Hubei Province. Despite this, limited by the lack of background information, we could not completely exclude the possibility that these strains were originally from one pig farm and spread by live herd transportation.

The deletion of amino acids 247–256 in GP2 occurs in Henan-A12, Henan-A13, Henan-A14, and MY-486, which provide evidence that deletion could occur in a relatively closed circumstance. These strains were isolated from a single pig farm between 2013 and 2014 by our group, and the deletion did not occur in strains Henan-A1 to Henan-A10, which were isolated earlier. The same situation might also happen in the deletion of NSP2 464-468aa (HeN1401 and HeN1601). The two strains were isolated in Henan province by the same group in different years; deletion in QY2010, QYYZ, GM2, and GD-KP, which were all isolated in Guangdong province in different[vanji] years (Table S1).

The influence of virulence by deletion, mutation, or insertion have been studied, and both positive and negative results were found. These results exhibited the complication of PRRSV genome change, and more data need to be collected in the future (Zhou et al., 2009; Zhao et al., 2018). Based on the PRRSV epidemic data discussed above, deletion, insertion, and mutation may drive virus evolution independently in a relatively closed or in a low strain diversity environment.

Recombination has been an important characteristic of PRRSV epidemics after 2006. The lower rate of recombination in 2006–2011 indicated that HP-PRRSV gained stronger capacity for recombination than Classic NA. However, either because its recombination ability was relatively weak (compared with NADC30) or because the pandemic strains had all been HP-PRRSV, the genetic status of PRRSV epidemics was still simple. A medium recombination rate was detected between 2012 and 2014, which may have arisen because the PRRSV epidemic situation was complicated by the emergence of the invasion of NADC30, which has strong recombination capacities. The dramatic increase in the recombination rate since 2015 may be attributable to the wide spread of NADC30-like strains (Figure 2; Yu et al., 2012; Sui et al., 2018; Zhou et al., 2018a,c; Zhang H. et al., 2019). It was difficult to identify the characteristic regions of recombination in the PRRSV genome in this research and other studies (Yuan et al., 1999; Wang L. J. et al., 2016; Liu et al., 2017; Zhou et al., 2018b,c; Zhang H. et al., 2019; Zhang W. L. et al., 2019). Evidences showed that recombination can changefv PRRSV virulence. For instance, strains JL580 and NADC30 both belong to the NADC30-like subgroup, but JL580 is a highly pathogenic virus, whereas NADC30 is a moderately virulent strain. The major difference in the two viruses is that a recombination event occurred in JL580; a difference in virulence was also detected between QY2010 and GM2. QY2010 is a highly pathogenic PRRSV strain (30% mortality), whereas its generation strain GM2 is a recombinant strain of QY2010 and RespPRRS MLV, which only causes 2.2% mortality in the field, and several other studies have reported the effects of recombination (Brockmeier et al., 2012; Deng et al., 2012; Wenhui et al., 2012;[vanji]Liu et al., 2018).

Our results indicate that recombination can occur between strains that belong to different subgroups, and that the Original HP-PRRSV and NADC30-like strains are very active in recombination (Figure 4 and Table 2). Because of the lack of virulence information of isolates, it is difficult to identify the role of recombination in virulence changes, which makes understanding the epidemic status of PRRSV more difficult. The results for isolates belonging to the Extensional and Propagating recombination subgroups indicate that recombination has strongly driven the evolution of PRRSV. Indeed, strains belonging to the Intermediate PRRSV subgroup mainly arose through recombination after 2015. The isolates from the Extensional and Propagating groups, especially the Propagating group, indicated that PRRSV could adapt to their circumstance by recombination, and recombination was the main driving force of PRRSV evolution. This should be taken into account during PRRS control in the future (Figures 4, 5).

The use of HP-PRRSV live vaccine was criticized after our group reported its virulence reversion, and controversy was also found between different results of immune efficiency against NADC30-like strain challenge (Jiang et al., 2015; Zhang et al., 2016; Zhou et al., 2017; Sun et al., 2018). The influence of Classic NA and HP-PRRSV live vaccine on virus evolution was discussed above.

It is difficult to assess the influence of the HP-PRRSV vaccine comprehensively, but some trends can be identified according to the result. Before 2006, vaccination was not widely used in China, and PRRSV was still evolving in its own way. From 2006 to 2010, the evolution of PRRSV was slow and simple. A new subgroup, PRRSV2010, emerged, which might be attributable to the use of the Classic NA vaccine and the killed HP-PRRSV vaccine in 2007 (http://www.moa.gov.cn/gk/tzgg_1/tz/200704/t20070403_795780.htm), raising concerns about the incomplete protective efficacy of the Classic NA vaccine and the killed vaccine against HP-PRRSV (Scortti et al., 2007; Lager et al., 2014). The isolate that belonged to the PRRSV2010 subgroup may have arisen because of the immune pressure exerted by inefficient immunization, and it disappeared 2 years after the use of the HP-PRRSV live vaccine. The use of the live HP-PRRSV vaccine actually limited the diversity of PRRSV from 2011 to 2014 by eradicating the wild strains of Classic NA and PRRSV2010 and limiting the transmission of the NADC30-like strains, if without regarding virulence reversion (Figure 2). There was an increased number of NADC30-like strain, Intermediate strain, and an increased rate of recombination after 2015 accompanied with a reduced use of the HP-PRRSV live vaccine. From this perspective, the HP-PRRSV live vaccine still provides protection against PRRSV and contributes to PRRS control, but the risk of virulence reversion must be considered, and the scientific use of the vaccine should be given the highest priority.

## Conclusion

The total PRRSV isolates in China could be divided into eight subgroups, and strains belonging to the Original HP-PRRSV, NADC30-like, and Intermediate PRRSV subgroups will play a major role in PRRS epidemics in the future, especially those recombinant strains that have integrated the advantages of their parental strains. Deletion was the main driving force of PRRSV evolution before 2006 and might have also contributed to the virus evolution in a relatively closed environment or low strain diversity area. However, recombination has played a very important role in virus evolution after 2006 and may cause considerable difficulty in PRRS control in the future.

## Data Availability Statement

The raw data supporting the conclusions of this article will be made available by the authors, without undue reservation, to any qualified researcher.

## Author Contributions

YJ wrote and collected the data. GL collected data and supervised the study. LY, LL, YZ, WT, and CL provided advice. YZ supervised and provided advice. FG and GT funded and supervised the study.

## Conflict of Interest

The authors declare that the research was conducted in the absence of any commercial or financial relationships that could be construed as a potential conflict of interest.

## References

[B1] Akay C. Thomas C 3rd Gazitt Y. (2004). Arsenic trioxide and paclitaxel induce apoptosis by different mechanisms. Cell Cycle 3, 324–334. 10.4161/cc.3.3.657 14726646

[B2] An T. Q. Tian Z. J. Xiao Y. Li R. Peng J. M. Wei T. C.. (2010). Origin of highly pathogenic porcine reproductive and respiratory syndrome virus, China. Emerging Infect. Dis. 16, 365–367. 10.3201/eid1602.090005 20113592 PMC2957991

[B3] Bautista E. M. Meulenberg J. J. Choi C. S. Molitor T. W. (1996). Structural polypeptides of the American (VR-2332) strain of porcine reproductive and respiratory syndrome virus. Arch. Virol. 141, 1357–1365. 10.1007/BF01718837 8774694

[B4] Benfield D. A. Nelson E. Collins J. E. Harris L. Goyal S. M. Robison D.. (1992). Characterization of swine infertility and respiratory syndrome (SIRS) virus (isolate ATCC VR-2332). J. Vet. Diagn. Invest. 4, 127–133. 10.1177/104063879200400202 1616976

[B5] Brockmeier S. L. Loving C. L. Vorwald A. C. Kehrli M. E. Jr. Baker R. B. Nicholson T. L.. (2012). Genomic sequence and virulence comparison of four type 2 porcine reproductive and respiratory syndrome virus strains. Virus Res. 169, 212–221. 10.1016/j.virusres.2012.07.030 23073232

[B6] Chen J. Z. Peng J. M. Bai Y. Wang Q. Liu Y. M. Zhang Q. Y.. (2015). Characterization of two novel porcine reproductive and respiratory syndrome virus isolates with deletions in the GP2 gene. Vet. Microbiol. 176, 344–351. 10.1016/j.vetmic.2015.01.018 25669596

[B7] Deng Y. Pan Y. Wang D. Zhou Q. Bi Y. Chen F.. (2012). Complete genome sequence of porcine reproductive and respiratory syndrome virus strain QY2010 reveals a novel subgroup emerging in China. J. Virol. 86, 7719–7720. 10.1128/JVI.00977-12 22733883 PMC3416273

[B8] Dong J. Wang Y. Yu L. Zhang P. Liu X. Zhang L.. (2017). Pathogenicity of a newly emerged recombined porcine reproductive and respiratory syndrome virus strain (subgenotype III) in China. Vet. Microbiol. 210, 162–166. 10.1016/j.vetmic.2017.09.018 29103686

[B9] Firth A. E. Zevenhoven-Dobbe J. C. Wills N. M. Go Y. Y. Balasuriya U. B. R. Atkins J. F.. (2011). Discovery of a small arterivirus gene that overlaps the GP5 coding sequence and is important for virus production. J. Gen. Virol. 92, 1097–1106. 10.1099/vir.0.029264-0 21307223 PMC3139419

[B10] Guo A. Wu G. Gong W. Luo X. Zheng H. Jia H.. (2012). Outbreaks of highly pathogenic porcine reproductive and respiratory syndrome in Jiangxi province, China. Ir. Vet. J. 65, 14. 10.1186/2046-0481-65-14 22784793 PMC3443052

[B11] Guo Z. Chen X. X. Li X. Qiao S. Deng R. Zhang G. (2019). Prevalence and genetic characteristics of porcine reproductive and respiratory syndrome virus in central China during 2016-2017: NADC30-like PRRSVs are predominant. Microb. Pathog. 135, 103657. 10.1016/j.micpath.2019.103657 31398529

[B12] Han W. Wu J. J. Deng X. Y. Cao Z. Yu X. L. Wang C. B.. (2009). Molecular mutations associated with the *in vitro* passage of virulent porcine reproductive and respiratory syndrome virus. Virus Genes 38, 276–284. 10.1007/s11262-008-0322-1 19132524

[B13] Jantafong T. Sangtong P. Saenglub W. Mungkundar C. Romlamduan N. Lekchareonsuk C.. (2015). Genetic diversity of porcine reproductive and respiratory syndrome virus in Thailand and Southeast Asia from 2008 to 2013. Vet. Microbiol. 176, 229–238. 10.1016/j.vetmic.2015.01.017 25704227

[B14] Jiang Y. F. Xia T. Q. Zhou Y. J. Yu L. X. Yang S. Huang Q. F.. (2015). Characterization of three porcine reproductive and respiratory syndrome virus isolates from a single swine farm bearing strong homology to a vaccine strain. Vet. Microbiol. 179, 242–249. 10.1016/j.vetmic.2015.06.015 26162970

[B15] Johnson C. R. Griggs T. F. Gnanandarajah J. Murtaugh M. P. (2011). Novel structural protein in porcine reproductive and respiratory syndrome virus encoded by an alternative ORF5 present in all arteriviruses. J. Gen. Virol. 92, 1107–1116. 10.1099/vir.0.030213-0 21307222 PMC3139420

[B16] Kvisgaard L. K. Larsen L. E. Hjulsager C. K. Botner A. Rathkjen P. H. Heegaard P. M. H.. (2017). Genetic and biological characterization of a Porcine Reproductive and Respiratory Syndrome Virus 2 (PRRSV-2) causing significant clinical disease in the field. Vet. Microbiol. 211, 74–83. 10.1016/j.vetmic.2017.10.001 29102125

[B17] Lager K. M. Schlink S. N. Brockmeier S. L. Miller L. C. Henningson J. N. Kappes M. A.. (2014). Efficacy of Type 2 PRRSV vaccine against Chinese and Vietnamese HP-PRRSV challenge in pigs. Vaccine 32, 6457–6462. 10.1016/j.vaccine.2014.09.046 25285886

[B18] Lassalle B. Bastos H. Louis J. P. Riou L. Testart J. Dutrillaux B.. (2004). ‘Side Population' cells in adult mouse testis express Bcrp1 gene and are enriched in spermatogonia and germinal stem cells. Development 131, 479–487. 10.1242/dev.00918 14681185

[B19] Leng X. Li Z. Xia M. He Y. Wu H. (2012). Evaluation of the efficacy of an attenuated live vaccine against highly pathogenic porcine reproductive and respiratory syndrome virus in young pigs. Clin. Vaccine Immunol. 19, 1199–1206. 10.1128/CVI.05646-11 22695163 PMC3416087

[B20] Li B. Fang L. Xu Z. Liu S. Gao J. Jiang Y.. (2009). Recombination in vaccine and circulating strains of porcine reproductive and respiratory syndrome viruses. Emerging Infect. Dis. 15, 2032–2035. 10.3201/eid1512.090390 19961694 PMC3044526

[B21] Liu J. Wei C. Lin Z. Xia W. Ma Y. Dai A.. (2019). Full genome sequence analysis of a 1-7-4-like PRRSV strain in Fujian Province, China. Peer J. 7:e7859. 10.7717/peerj.7859 31637126 PMC6800524

[B22] Liu J. Zhou X. Zhai J. Wei C. Dai A. Yang X.. (2017). Recombination in JXA1-R vaccine and NADC30-like strain of porcine reproductive and respiratory syndrome viruses. Vet. Microbiol. 204, 110–120. 10.1016/j.vetmic.2017.04.017 28532789

[B23] Liu J. K. Wei C. H. Yang X. Y. Hou X. L. Dai A. L. Li X. H.. (2013). Genetic diversity and evolutionary characterization of Chinese porcine reproductive and respiratory syndrome viruses based on NSP2 and ORF5. Arch. Virol. 158, 1811–1816. 10.1007/s00705-013-1669-8 23525729

[B24] Liu Y. Li J. Yang J. Zeng H. Guo L. Ren S. (2018). Emergence of different recombinant porcine reproductive and respiratory syndrome viruses, China. Sci. Rep. 8:4118 10.1038/s41598-018-22494-4 29515183 PMC5841431

[B25] Lu W. H. Tun H. M. Sun B. L. Mo J. Zhou Q. F. Deng Y. X.. (2015). Re-emerging of porcine respiratory and reproductive syndrome virus (lineage 3) and increased pathogenicity after genomic recombination with vaccine variant. Vet. Microbiol. 175, 332–340. 10.1016/j.vetmic.2014.11.016 25529828

[B26] Meulenberg J. J. Petersen Den Besten A. de Kluyver E. van Nieuwstadt A. Wensvoort G. Moormann R. J. (1997). Molecular characterization of Lelystad virus. Vet. Microbiol. 55, 197–202. 10.1016/S0378-1135(96)01335-1 9220614 PMC7117323

[B27] Nelsen C. J. Murtaugh M. P. Faaberg K. S. (1999). Porcine reproductive and respiratory syndrome virus comparison: divergent evolution on two continents. J. Virol. 73, 270–280. 10.1128/JVI.73.1.270-280.1999 9847330 PMC103831

[B28] Neumann E. J. Kliebenstein J. B. Johnson C. D. Mabry J. W. Bush E. J. Seitzinger A. H.. (2005). Assessment of the economic impact of porcine reproductive and respiratory syndrome on swine production in the United States. J. Am. Vet. Med. Assoc. 227, 385–392. 10.2460/javma.2005.227.385 16121604

[B29] Ostrowski M. Galeota J. A. Jar A. M. Platt K. B. Osorio F. A. Lopez O. J. (2002). Identification of neutralizing and nonneutralizing epitopes in the porcine reproductive and respiratory syndrome virus GP5 ectodomain. J. Virol. 76, 4241–4250. 10.1128/JVI.76.9.4241-4250.2002 11932389 PMC155073

[B30] Scortti M. Prieto C. Alvarez E. Simarro I. Castro J. M. (2007). Failure of an inactivated vaccine against porcine reproductive and respiratory syndrome to protect gilts against a heterologous challenge with PRRSV. Vet. Rec. 161, 809–813. 10.1136/vr.161.24.809 18083979

[B31] Shi M. Holmes E. C. Brar M. S. Leung F. C. (2013). Recombination is associated with an outbreak of novel highly pathogenic porcine reproductive and respiratory syndrome viruses in China. J. Virol. 87, 10904–10907. 10.1128/JVI.01270-13 23885071 PMC3807407

[B32] Snijder E. J. Meulenberg J. J. (1998). The molecular biology of arteriviruses. J. Gen. Virol. 79 (Pt. 5), 961–979. 10.1099/0022-1317-79-5-961 9603311

[B33] Snijder E. J. Wassenaar A. L. Spaan W. J. (1994). Proteolytic processing of the replicase ORF1a protein of equine arteritis virus. J. Virol. 68, 5755–5764. 10.1128/JVI.68.9.5755-5764.1994 8057457 PMC236979

[B34] Suarez P. Zardoya R. Martin M. J. Prieto C. Dopazo J. Solana A.. (1996). Phylogenetic relationships of european strains of porcine reproductive and respiratory syndrome virus (PRRSV) inferred from DNA sequences of putative ORF-5 and ORF-7 genes. Virus Res. 42, 159–165. 10.1016/0168-1702(95)01305-9 8806183

[B35] Sui X. Guo X. Jia H. Wang X. Lin W. Li M.. (2018). Genomic sequence and virulence of a novel NADC30-like porcine reproductive and respiratory syndrome virus isolate from the Hebei province of China. Microb. Pathog. 125, 349–360. 10.1016/j.micpath.2018.08.048 30149129

[B36] Sun Y. F. Zhou L. Bian T. Tian X. X. Ren W. K. Lu C.. (2018). Efficacy evaluation of two commercial modified-live virus vaccines against a novel recombinant type 2 porcine reproductive and respiratory syndrome virus. Vet. Microbiol. 216, 176–182. 10.1016/j.vetmic.2018.02.016 29519513

[B37] Tian Z. J. An T. Q. Zhou Y. J. Peng J. M. Hu S. P. Wei T. C.. (2009). An attenuated live vaccine based on highly pathogenic porcine reproductive and respiratory syndrome virus (HP-PRRSV) protects piglets against HP-PRRS. Vet. Microbiol. 138, 34–40. 10.1016/j.vetmic.2009.03.003 19339125

[B38] Tong G. Z. Zhou Y. J. Hao X. F. Tian Z. J. An T. Q. Qiu H. J. (2007). Highly pathogenic porcine reproductive and respiratory syndrome, China. Emerging Infect. Dis. 13, 1434–1436. 10.3201/eid1309.070399 18252136 PMC2857295

[B39] Valicek L. Psikal I. Smid B. Rodak L. Kubalikova R. Kosinova E. (1997). Isolation and identification of porcine reproductive and respiratory syndrome virus in cell cultures. Vet. Med. (Praha). 42, 281–287. 9416008

[B40] van Dinten L. C. Wassenaar A. L. Gorbalenya A. E. Spaan W. J. Snijder E. J. (1996). Processing of the equine arteritis virus replicase ORF1b protein: identification of cleavage products containing the putative viral polymerase and helicase domains. J. Virol. 70, 6625–6633. 10.1128/JVI.70.10.6625-6633.1996 8794297 PMC190703

[B41] Wang F. X. Qin L. T. Liu Y. Liu X. Sun N. Yang Y.. (2015). Novel Nsp2 deletion based on molecular epidemiology and evolution of porcine reproductive and respiratory syndrome virus in Shandong Province from 2013 to 2014. Infect. Genet. Evol. 33, 219–226. 10.1016/j.meegid.2015.05.006 25958135

[B42] Wang L. J. Guo Z. Qiao S. Chen X. X. Zhang G. (2016). Complete genome sequence of a mosaic NADC30-like porcine reproductive and respiratory syndrome virus in China. Genome Announc. 4. 10.1128/genomeA.01428-16 28007861 PMC5180389

[B43] Wang L. J. Wan B. Guo Z. Qiao S. Li R. Xie S.. (2018). Genomic analysis of a recombinant NADC30-like porcine reproductive and respiratory syndrome virus in china. Virus Genes 54, 86–97. 10.1007/s11262-017-1516-1 29090410

[B44] Wang X. Yang X. Zhou R. Zhou L. Ge X. Guo X.. (2016). Genomic characterization and pathogenicity of a strain of type 1 porcine reproductive and respiratory syndrome virus. Virus Res. 225, 40–49. 10.1016/j.virusres.2016.09.006 27619842

[B45] Wenhui L. Zhongyan W. Guanqun Z. Zhili L. Jingyun M. Qingmei X.. (2012). Complete genome sequence of a novel variant porcine reproductive and respiratory syndrome virus (PRRSV) strain: evidence for recombination between vaccine and wild-type PRRSV strains. J. Virol. 86:9543. 10.1128/JVI.01341-12 22879614 PMC3416112

[B46] Wensvoort G. De Kluyver E. P. Pol J. M. Wagenaar F. Moormann R. J. Hulst M. M.. (1992). Lelystad virus, the cause of porcine epidemic abortion and respiratory syndrome: a review of mystery swine disease research at Lelystad. Vet. Microbiol. 33, 185–193. 10.1016/0378-1135(92)90046-V 1481355

[B47] Xia J. Chen S. Huang J. Ma W. Du W. Yang X.. (2015). Complete genomic characterization of a porcine reproductive and respiratory syndrome virus isolate in Xinjiang province of China. Virus Genes 50, 39–45. 10.1007/s11262-014-1122-4 25272960

[B48] Xie J. Zhu W. Chen Y. Wei C. Zhou P. Zhang M.. (2013). Molecular epidemiology of PRRSV in South China from 2007 to 2011 based on the genetic analysis of ORF5. Microb. Pathog. 63, 30–36. 10.1016/j.micpath.2013.05.013 23770054

[B49] Yan Y. Xin A. Zhu G. Huang H. Liu Q. Shao Z. (2013). Complete genome sequence of a novel natural recombinant porcine reproductive and respiratory syndrome virus isolated from a pig farm in yunnan province, southwest china. Genome Announc. 1:e0010113 10.1128/genomeA.00101-13 23405309 PMC3569298

[B50] Yu X. Chen N. Wang L. Wu J. Zhou Z. Ni J. (2012). New genomic characteristics of highly pathogenic porcine reproductive and respiratory syndrome viruses do not lead to significant changes in pathogenicity. Vet. Microbiol. 158, 291–299. 10.1016/j.vetmic.2012.02.036 22525010

[B51] Yuan S. Nelsen C. J. Murtaugh M. P. Schmitt B. J. Faaberg K. S. (1999). Recombination between North American strains of porcine reproductive and respiratory syndrome virus. Virus Res. 61, 87–98. 10.1016/S0168-1702(99)00029-5 10426212 PMC7125646

[B52] Zhang H. Leng C. Ding Y. Zhai H. Li Z. Xiang L.. (2019). Characterization of newly emerged NADC30-like strains of porcine reproductive and respiratory syndrome virus in China. Arch. Virol. 164, 401–411. 10.1007/s00705-018-4080-7 30353281

[B53] Zhang Q. Bai J. Hou H. Song Z. Zhao Y. Jiang P. (2017). A novel recombinant porcine reproductive and respiratory syndrome virus with significant variation in cell adaption and pathogenicity. Vet. Microbiol. 208, 150–158. 10.1016/j.vetmic.2017.07.028 28888630

[B54] Zhang Q. Jiang P. Song Z. Lv L. Li L. Bai J. (2016). Pathogenicity and antigenicity of a novel NADC30-like strain of porcine reproductive and respiratory syndrome virus emerged in China. Vet. Microbiol. 197, 93–101. 10.1016/j.vetmic.2016.11.010 27938690

[B55] Zhang W. L. Zhang H. L. Xu H. Tang Y. D. Leng C. L. Peng J. M.. (2019). Two novel recombinant porcine reproductive and respiratory syndrome viruses belong to sublineage 3.5 originating from sublineage 3.2. Transbound. Emerg. Dis. 66, 2592–2600. 10.1111/tbed.13320 31379138

[B56] Zhao K. Gao J. C. Xiong J. Y. Guo J. C. Yang Y. B. Jiang C. G.. (2018). Two residues in NSP9 contribute to the enhanced replication and pathogenicity of highly pathogenic porcine reproductive and respiratory syndrome virus. J. Virol. 92. 10.1128/JVI.02209-17 29321316 PMC5972891

[B57] Zhao D. Liu R. Zhang X. Li F. Wang J. Zhang J.. (2019). Replication and virulence in pigs of the first African swine fever virus isolated in China. Emerg. Microbes Infect. 8, 438–447. 10.1080/22221751.2019.1590128 30898043 PMC6455124

[B58] Zhao H. Han Q. Zhang L. Zhang Z. Wu Y. Shen H.. (2017). Emergence of mosaic recombinant strains potentially associated with vaccine JXA1-R and predominant circulating strains of porcine reproductive and respiratory syndrome virus in different provinces of China. Virol. J. 14:67. 10.1186/s12985-017-0735-3 28376821 PMC5379541

[B59] Zhao K. Ye C. Chang X. B. Jiang C. G. Wang S. J. Cai X. H.. (2015). Importation and recombination are responsible for the latest emergence of highly pathogenic porcine reproductive and respiratory syndrome virus in China. J. Virol. 89, 10712–10716. 10.1128/JVI.01446-15 26246582 PMC4580157

[B60] Zhou L. Kang R. Xie B. Tian Y. Wu X. Lv X.. (2018b). Identification of a novel recombinant type 2 porcine reproductive and respiratory syndrome virus in China. Viruses 10. 10.3390/v10040151 29584650 PMC5923445

[B61] Zhou L. Kang R. Ji G. Tian Y. Ge M. Xie B.. (2018a). Molecular characterization and recombination analysis of porcine reproductive and respiratory syndrome virus emerged in southwestern China during 2012-2016. Virus Genes 54, 98–110. 10.1007/s11262-017-1519-y 29138994

[B62] Zhou L. Kang R. Xie B. Tian Y. Yang X. Yu J.. (2018c). Complete Genome sequence of a recombinant NADC30-like strain, SCnj16, of porcine reproductive and respiratory syndrome virus in southwestern China. Genome Announc. 6, e00004–e00018. 10.1128/genomeA.00004-18 29439029 PMC5805867

[B63] Zhou L. Yang B. Xu L. Jin H. Ge X. Guo X.. (2017). Efficacy evaluation of three modified-live virus vaccines against a strain of porcine reproductive and respiratory syndrome virus NADC30-like. Vet. Microbiol. 207, 108–116. 10.1016/j.vetmic.2017.05.031 28757009

[B64] Zhou L. Yang H. (2010). Porcine reproductive and respiratory syndrome in China. Virus Res. 154, 31–37. 10.1016/j.virusres.2010.07.016 20659506

[B65] Zhou L. Zhang J. Zeng J. Yin S. Li Y. Zheng L. (2009). The 30-amino-acid deletion in the Nsp2 of highly pathogenic porcine reproductive and respiratory syndrome virus emerging in China is not related to its virulence. J. Virol. 83, 5156–5167. 10.1128/JVI.02678-08 19244318 PMC2682102

[B66] Zhou Y. J. Hao X. F. Tian Z. J. Tong G. Z. Yoo D. An T. Q.. (2008). Highly virulent porcine reproductive and respiratory syndrome virus emerged in China. Transbound. Emerg. Dis. 55, 152–164. 10.1111/j.1865-1682.2008.01020.x 18405338

