# Supplementary material for: Genetic Diversity of Porcine Reproductive and Respiratory Syndrome Virus (PRRSV) From 1996 to 2017 in China
Source: Front Microbiol. 2020 Apr 24;11:618. doi: 10.3389/fmicb.2020.00618 (PMC7193098; doi:10.3389/fmicb.2020.00618)
Supplement: Supplementary file 1 [file Table_1.docx]

Table S1 Information of PRRSV Strains used in this study

|  | Name | NO. | Isolate/collection date | Country | Characteristic | |
| --- | --- | --- | --- | --- | --- | --- |
|  |  |  |  |  | Deletion | Insertion |
| 1 | Lelystad virus(LV) | M96262 | 1991 | Netherlands | / | / |
| 2 | GZ11-G1 | KF001144 | 2011 | China | / | / |
| 3 | 15HEN1_EU | KX967492 | 2015 | China | / | / |
| 4 | LNEU12 | KM196101 | 2012 | China | / | / |
| 5 | BJEU06-1 | GU047344 | 2006 | China | / | / |
| 6 | NVDC-NM1-2011 | JX187609 | 2011 | China | / | / |
| 7 | NMEU09-1 | GU047345 | 2009 | China | / | / |
| 8 | NVDC-NM2 | KC492504 | 2011 | China | / | / |
| 9 | NVDC-FJ | KC492506 | 2011 | China | / | / |
| 10 | NVDC-NM3 | KC492505 | 2011 | China | / | / |
| 11 | lena | JF802085 | 2007 | Belgium | / | / |
| 12 | VR2332 | EF536003 | 1992 | USA | - | - |
| 13 | Ingelvac ATP | DQ988080 | - | USA | - | - |
| 14 | HB-2(sh)/2002 | AY262352 | 2002 | China | NSP2 472-483aa, GP5a 47-51aa, GP3 100aa | - |
| 15 | CH-1R | EU807840 | - | China | NSP2 631aa, GP5a 47-51aa | - |
| 16 | HH08 | JX679179 | 2008 | China | NSP2 631aa, GP5a 47-51aa | - |
| 17 | CH-1a | AY032626 | 1996 | China | GP5a 47-51aa | - |
| 18 | CH2004 | EU880439 | 2004 | China | GP5a 47-51aa | - |
| 19 | CH2002 | EU880438 | 2002 | China | GP5a 47-51aa | - |
| 20 | CH2003 | EU880440 | 2003 | China | GP5a 47-51aa | - |
| 21 | GZ1101 | KF771273 | 2011 | China | NSP2 594-596aa, GP5 34aa, GP5a 36aa, | - |
| 22 | HN1 | AY457635 | 2003 | China | - | - |
| 23 | PRRSV02 | FJ175688 | 2002 | China | - | - |
| 24 | PRRSV03 | FJ175689 | 2003 | China | - | - |
| 25 | RespPRRS MLV | AF066183 | - | USA | - | - |
| 26 | DY | JN864948 | 2007 | China | - | - |
| 27 | YN-2011 | JX857698 | 2011 | China | - | - |
| 28 | CC-1 | EF153486 | 2006 | China | - | - |
| 29 | Clone20 | FJ899592 | 2003 | China | - | - |
| 30 | SD1-100 | GQ914997 | 2009 | China | - | - |
| 31 | PRRSV01 | FJ175687 | 2001 | China | - | - |
| 32 | S1 | DQ459471 | 1998 | China | - | - |
| 33 | BJ-4 | AF331831 | 1996 | China | NSP2 697aa | - |
| 34 | GS2004 | EU880443 | 2004 | China | NSP2 697aa | - |
| 35 | GS2002 | EU880441 | 2002 | China | NSP2 697aa | - |
| 36 | GS2003 | EU880442 | 2003 | China | NSP2 697aa | - |
| 37 | Em2007 | EU262603 | 2007 | China | NSP2 499-566aa, GP5a 47-51aa | - |
| 38 | GD-KP | KU978619 | 2015 | China | NSP2 301-302aa,468-512aa, 540-568aa, | NSP2 818-853aa |
| 39 | GM2 | JN662424 | 2011 | China | NSP2 301-302aa | NSP2 818-853aa |
| 40 | QY2010 | JQ743666 | 2010 | China | NSP2 301-302aa | NSP2 818-853aa |
| 41 | QYYZ | JQ308798 | 2011 | China | NSP2 301-302aa | NSP2 818-853aa |
| 42 | ZJXS1412 | MF669722 | 2014 | China | NSP2 482aa 534-562aa,GP5a 47-51aa | - |
| 43 | HeN1401 | MF766471 | 2014 | China | NSP2 324-434aa 464-468aa 486aa 502-520aa | - |
| 44 | HeN1601 | MF766474 | 2016 | China | NSP2 324-434aa 464-468aa 486aa 502-520aa | - |
| 45 | SDhz1512 | KX980392 | 2015 | China | NSP2 482aa 534-562aa | - |
| 46 | SDlz1601 | KX980393 | 2016 | China | NSP2 324-434aa 486aa 502-520aa, GP5a 47-51aa | - |
| 47 | HZ-31 | KC445138 | 2012 | China | NSP2 468-497aa 534-562aa | - |
| 48 | GDZS2016 | MH046843 | 2016 | China | NSP2 482aa 534-562aa,GP5a 47-51aa | - |
| 49 | SCya17 | MH324400 | 2017 | China | NSP2 482aa 534-562aa,GP5a 47-51aa | - |
| 50 | GDQYQC2 | MF526896 | 2016 | China | NSP2 482aa 534-562aa,GP5a 36aa, GP5 37aa | - |
| 51 | GDYDZZZ | KY745901 | 2016 | China | NSP2 482aa 534-562aa,GP5a 39aa, GP5 37aa | - |
| 52 | HZL1501 | MF669721 | 2015 | China | NSP2 16-25aa 482aa 534-562aa, | - |
| 53 | XJzx1-2015 | KX689233 | 2015 | China | NSP2 469-473aa,GP5a 47-51aa, GP2 173-174aa | - |
| 54 | SH1211 | KF678434 | 2012 | China | NSP2 482aa 534-562aa,GP3 68-69aa | - |
| 55 | HLJB1 | KT351740 | 2013 | China | - | - |
| 56 | NJ-1106 | JX880029 | 2011 | China | NSP2 482aa 534-562aa, 621-764aa, GP5a 47-51aa | - |
| 57 | NT0801 | HQ315836 | 2008 | China | GP5a 47-51aa | - |
| 58 | BJ0706 | GQ351601 | 2007 | China | NSP2 482aa, GP5a 47-51aa | - |
| 59 | GD3 | GU269541 | 2005 | China | NSP2 482aa, GP5a 47-51aa | - |
| 60 | NB/04 | FJ536165 | 2004 | China | NSP2 482aa, GP5a 47-51aa | - |
| 61 | YN-1 | KJ747052 | 2011 | China | NSP2 534-562aa, GP5a 47-51aa | - |
| 62 | SHB | EU864232 | 2005 | China | GP5a 47-51aa | - |
| 63 | HB-1(sh)/2002 | AY150312 | 2002 | China | GP5a 47-51aa | - |
| 64 | LN1101 | KF751238 | 2011 | China | NSP2 483-500aa,GP5a 47-51aa | - |
| 65 | NVDC-BJ2-2011 | KP771748 | 2011 | China | NSP2 482aa 534-562aa, GP5a 47-51aa | - |
| 66 | ZCYZ | JF800911 | 2009 | China | NSP2 475-499aa 534-562aa, GP5a 47-51aa | - |
| 67 | NVDC-GD2-2011 | JQ715697 | 2011 | China | NSP2 477-489aa 534-562aa, GP5a 47-51aa | - |
| 68 | HN-HW | FJ797690 | 2006 | China | NSP2 482aa 534-562aa,GP5a 47-51aa | - |
| 69 | HUN4 | EF635006 | 2006 | China | NSP2 482aa 534-562aa,GP5a 47-51aa | - |
| 70 | GDQJ | GQ374441 | 2007 | China | NSP2 482aa 534-562aa,GP5a 47-51aa | - |
| 71 | HN2007 | EU880437 | 2007 | China | NSP2 482aa 534-562aa,GP5a 47-51aa | - |
| 72 | GD2007 | EU880433 | 2007 | China | NSP2 482aa 534-562aa,GP5a 47-51aa | - |
| 73 | SC/NJ 2016 | MF818049 | 2016 | China | NSP2 482aa 534-562aa,GP5a 47-51aa | - |
| 74 | WUH4 | JQ326271 | 2011 | China | NSP2 482aa 534-562aa,GP5a 47-51aa | - |
| 75 | JX143 | EU708726 | 2006 | China | NSP2 482aa 534-562aa,GP5a 47-51aa | - |
| 76 | Jiangxi-3 | EU200961 | 2007 | China | NSP2 482aa 534-562aa,GP5a 47-51aa | - |
| 77 | FZ06A | MF370557 | 2006 | China | NSP2 482aa 534-562aa,GP5a 47-51aa | - |
| 78 | HEB1 | EF112447 | 2006 | China | NSP2 482aa 534-562aa,GP5a 47-51aa | - |
| 79 | 09HEB | JF268679 | 2009 | China | NSP2 482aa 534-562aa,GP5a 47-51aa | - |
| 80 | SX-1 | GQ857656 | 2009 | China | NSP2 482aa 534-562aa,GP5a 47-51aa | - |
| 81 | Henan-1 | EU200962 | 2007 | China | NSP2 482aa 534-562aa,GP5a 47-51aa | - |
| 82 | BJ | EU825723 | 2007 | China | NSP2 482aa 534-562aa,GP5a 47-51aa | - |
| 83 | QH-08 | KU201579 | 2008 | China | NSP2 616-645aa, GP5a 47-51aa | - |
| 84 | HENZZ-8 | KU950375 | 2015 | China | NSP2 482aa 534-562aa,GP5a 47-51aa | - |
| 85 | NVDC-SC1-2014 | KP771739 | 2014 | China | NSP2 482aa 534-562aa,GP5a 47-51aa | - |
| 86 | 10-10QN | JQ663556 | 2010 | China | NSP2 482aa 534-562aa,GP5a 47-51aa | - |
| 87 | SCwhn14DY | KT819203 | 2014 | China | NSP2 468-503aa 534-562aa, GP5a 47-51aa | - |
| 88 | BJBLZ | FJ950745 | 2007 | China | NSP2 482aa 534-562aa,GP5a 47-51aa | - |
| 89 | HV | JX317648 | 2007 | China | NSP2 482aa 534-562aa,GP5a 47-51aa | - |
| 90 | SX2007 | EU880434 | 2007 | China | NSP2 482aa 534-562aa,GP5a 47-51aa | - |
| 91 | YN2008 | EU880435 | 2008 | China | NSP2 482aa 534-562aa,GP5a 47-51aa | - |
| 92 | GD | EU109503 | 2006 | China | NSP2 482aa 534-562aa,GP5a 47-51aa | - |
| 93 | GS2008 | EU880431 | 2008 | China | NSP2 482aa 534-562aa,GP5a 47-51aa | - |
| 94 | JSyx | EU939312 | 2006 | China | NSP2 482aa 534-562aa,GP5a 47-51aa | - |
| 95 | WUH1 | EU187484 | 2006 | China | NSP2 482aa 534-562aa,GP5a 47-51aa | - |
| 96 | GDBY1 | GQ374442 | 2008 | China | NSP2 482aa 534-562aa,GP5a 47-51aa | - |
| 97 | GD-HD | KP793736 | 2011 | China | NSP2 482aa 534-562aa,GP5a 47-51aa | - |
| 98 | 09HUN2 | JF268674 | 2009 | China | NSP2 482aa 534-562aa,GP5a 47-51aa | - |
| 99 | 10HN-GD | JX192632 | 2010 | China | NSP2 482aa 534-562aa,GP5a 47-51aa | - |
| 100 | NVDC-NM-2008 | KP771779 | 2008 | China | NSP2 472-501aa 534-562aa,GP5a 47-51aa | - |
| 101 | SY0608 | EU144079 | 2006 | China | NSP2 482aa 534-562aa,GP5a 47-51aa | - |
| 102 | 09HUN1 | JF268673 | 2009 | China | NSP2 482aa 534-562aa,GP5a 47-51aa | - |
| 103 | SCwhn09CD | JN836553 | 2009 | China | NSP2 15-21aa 482aa 534-562aa,GP5a 47-51aa | - |
| 104 | NVDC-CQ1-2011 | KP771746 | 2011 | China | NSP2 482aa 534-562aa,GP5a 47-51aa | - |
| 105 | NVDC-CQ3-2011 | KP771774 | 2011 | China | NSP2 482aa 534-562aa,GP5a 47-51aa | - |
| 106 | CBB-1-F3 | FJ889129 | 2008 | China | NSP2 482aa 534-562aa,GP5a 47-51aa | - |
| 107 | NVDC-CQ1-2012 | KP771747 | 2012 | China | NSP2 482aa 534-562aa,GP5a 47-51aa | - |
| 108 | NVDC-HeB1-2013 | KP771745 | 2013 | China | NSP2 474-522aa 534-562aa,GP5a 47-51aa | - |
| 109 | NVDC-HeB2-2013 | KP771744 | 2013 | China | NSP2 474-522aa 534-562aa,GP5a 47-51aa | - |
| 110 | GX09-16 | HM214913 | 2009 | China | NSP2 482aa 534-562aa,GP5a 47-51aa | - |
| 111 | CG | EU864231 | 2007 | China | NSP2 472-507aa 534-562aa,GP5a 47-51aa | - |
| 112 | GDQY2 | GU454850 | 2007 | China | NSP2 472-507aa 534-562aa,GP5a 47-51aa | - |
| 113 | GDQY1 | JN387271 | 2007 | China | NSP2 482aa 534-562aa,GP5a 47-51aa | - |
| 114 | TP | EU864233 | 2006 | China | NSP2 482aa 534-562aa,GP5a 47-51aa | - |
| 115 | YN9 | GU232738 | 2008 | China | NSP2 477-501aa 534-562aa,GP5a 47-51aa | - |
| 116 | 08SDWF | GU168569 | 2008 | China | NSP2 482aa 534-562aa,GP5a 47-51aa | - |
| 117 | HPBEDV | EU236259 | 2007 | China | NSP2 482aa 534-562aa,GP5a 47-51aa | - |
| 118 | JXA1 | EF112445 | 2006 | China | NSP2 482aa 534-562aa,GP5a 47-51aa | - |
| 119 | SHH | EU106888 | 2006 | China | NSP2 482aa 534-562aa,GP5a 47-51aa | - |
| 120 | JXwn06 | EF641008 | 2006 | China | NSP2 482aa 534-562aa,GP5a 47-51aa | - |
| 121 | TJ | EU860248 | 2006 | China | NSP2 482aa 534-562aa,GP5a 47-51aa | - |
| 122 | NVDC-HeB-2008 | KP771754 | 2008 | China | NSP2 482aa 534-562aa,GP5a 47-51aa | - |
| 123 | BJPG | FJ950746 | 2008 | China | NSP2 482aa 534-562aa,GP5a 47-51aa | - |
| 124 | LN | EU109502 | 2006 | China | NSP2 482aa 534-562aa,GP5a 47-51aa | - |
| 125 | NVDC-CQ-2008 | KP771755 | 2008 | China | NSP2 482aa 534-562aa,GP5a 47-51aa | - |
| 126 | HUB1 | EF075945 | 2006 | China | NSP2 482aa 534-562aa,GP5a 47-51aa | - |
| 127 | HUB2 | EF112446 | 2006 | China | NSP2 482aa 534-562aa,GP5a 47-51aa | - |
| 128 | SY0909 | HQ315837 | 2009 | China | NSP2 482aa 534-562aa,GP5a 47-51aa | - |
| 129 | AH0701 | GU461292 | 2007 | China | NSP2 482aa 534-562aa,GP5a 47-51aa | - |
| 130 | NVDC-CQ3-2012 | KP771775 | 2012 | China | NSP2 482aa 534-562aa,GP5a 47-51aa | - |
| 131 | SD-CXA2008 | GQ359108 | 2008 | China | NSP2 482aa 534-562aa,GP5a 47-51aa | - |
| 132 | HLJ-09 | HQ843178 | 2009 | China | NSP2 482aa 534-562aa,GP5a 47-51aa | - |
| 133 | SX-09 | HQ843181 | 2009 | China | NSP2 482aa 534-562aa,GP5a 47-51aa | - |
| 134 | HLM-09 | HQ843179 | 2009 | China | NSP2 482aa 534-562aa,GP5a 47-51aa | - |
| 135 | SD-09 | HQ843180 | 2009 | China | NSP2 482aa 534-562aa,GP5a 47-51aa | - |
| 136 | SD17 | MH404256 | 2017 | China | NSP2 482aa 534-562aa,GP5a 39aa 47-51aa | - |
| 137 | NVDC-HeB2-2012 | KP771772 | 2012 | China | NSP2 482aa 534-562aa,GP5a 47-51aa | - |
| 138 | JX | JX317649 | 2010 | China | NSP2 482aa 534-562aa,GP5a 47-51aa | - |
| 139 | GX09-29 | HM214914 | 2009 | China | NSP2 482aa 534-562aa,GP5a 47-51aa | - |
| 140 | 07QN | FJ394029 | 2007 | China | NSP2 482aa 534-562aa,GP2 255-256aa,GP5a 47-51aa | - |
| 141 | 08HuN | GU169411 | 2008 | China | NSP2 482aa 534-562aa,GP5a 47-51aa | - |
| 142 | GX09-32 | HM214915 | 2009 | China | NSP2 482aa 534-562aa,GP5a 47-51aa | - |
| 143 | BJsy06 | EU097707 | 2006 | China | NSP2 482aa 534-562aa,GP5a 47-51aa | - |
| 144 | NX06 | EU097706 | 2006 | China | NSP2 482aa 534-562aa,GP5a 47-51aa | - |
| 145 | JX2006 | EU880432 | 2006 | China | NSP2 482aa 534-562aa,GP5a 47-51aa | - |
| 146 | XL2008 | EU880436 | 2008 | China | NSP2 482aa 534-562aa,GP5a 47-51aa | - |
| 147 | Henan-A5 | KJ534540 | 2013 | China | NSP2 482aa 534-562aa,GP5a 47-51aa | - |
| 148 | Henan-A6 | KJ534541 | 2013 | China | NSP2 482aa 534-562aa,GP5a 47-51aa | - |
| 149 | Henan-A7 | KJ534542 | 2013 | China | NSP2 482aa 534-562aa,GP5a 47-51aa | - |
| 150 | Henan-A8 | KJ534543 | 2013 | China | NSP2 482aa 534-562aa,GP5a 47-51aa | - |
| 151 | 14LY01-FJ | KP780881 | 2014 | China | NSP2 324-434aa 486aa 502-520aa, GP5a 47-51aa | - |
| 152 | 15LY02-FJ | KU215417 | 2015 | China | NSP2 324-434aa 486aa 502-520aa, GP5a 47-51aa | - |
| 153 | 14LY02-FJ | KP780882 | 2014 | China | NSP2 324-434aa 486aa 502-520aa, GP5a 47-51aa | - |
| 154 | 15LY01-FJ | KU215416 | 2015 | China | NSP2 324-434aa 486aa 502-520aa, GP5a 47-51aa | - |
| 155 | HuN | EF517962 | 2006 | China | NSP2 482aa 534-562aa,GP5a 47-51aa | - |
| 156 | BJSY07 | HM011104 | 2007 | China | NSP2 482aa 534-562aa,GP5a 47-51aa | - |
| 157 | BJSY-1 | FJ950744 | 2007 | China | NSP2 482aa 534-562aa,GP5a 47-51aa | - |
| 158 | NVDC-BJ7-2012 | KP771758 | 2012 | China | NSP2 482aa 534-562aa,GP5a 47-51aa | - |
| 159 | NVDC-BJ8-2012 | KP771757 | 2012 | China | NSP2 482aa 534-562aa,GP5a 47-51aa | - |
| 160 | XH-GD | EU624117 | 2007 | China | NSP2 482aa 534-562aa,GP5a 47-51aa | - |
| 161 | 10GZ-GD | JX192633 | 2010 | China | NSP2 482aa 534-562aa,GP5a 47-51aa | - |
| 162 | 11GZ-GD | JX235370 | 2011 | China | NSP2 482aa 534-562aa,GP5a 47-51aa | - |
| 163 | 11FS11-GD | JX215551 | 2011 | China | NSP2 482aa 534-562aa,GP5a 47-51aa | - |
| 164 | 11FS12-GD | JX215554 | 2011 | China | NSP2 482aa 534-562aa,GP5a 47-51aa | - |
| 165 | CWZ-1-F3 | FJ889130 | 2008 | China | NSP2 482aa 534-562aa,GP5a 47-51aa | - |
| 166 | NVDC-CQ4-2012 | KP771777 | 2012 | China | NSP2 482aa 534-562aa,GP5a 47-51aa | - |
| 167 | NVDC-CQ2-2012 | KP771776 | 2012 | China | NSP2 482aa 534-562aa,GP5a 47-51aa | - |
| 168 | GD-P100 | GU143913 | - | China | NSP2 482aa 534-562aa,GP5a 47-51aa |  |
| 169 | 07BJ | FJ393459 | 2007 | China | NSP2 482aa 534-562aa,GP5a 47-51aa | - |
| 170 | GD | EU825724 | 2007 | China | NSP2 482aa 534-562aa,GP5a 47-51aa | - |
| 171 | 17-ZJ-HZ | MF770574 | 2017 | China | NSP2 482aa 534-562aa,GP5a 47-51aa | - |
| 172 | GD1404 | MF669720 | 2014 | China | NSP2 482aa 534-562aa 630-749aa,GP5a 47-51aa | - |
| 173 | XJu-1 | KF815525 | 2012 | China | NSP2 482aa 534-562aa,NSP2 630-749aa, GP5a 47-51aa | - |
| 174 | HeN1301 | MF766470 | 2013 | China | NSP2 482aa 534-562aa,GP5a 47-51aa | - |
| 175 | TJbd14-1 | KP742986 | 2014 | China | NSP2 482aa 534-562aa,NSP2 630-749aa, GP5a 47-51aa | - |
| 176 | TJbd14-2 | KP742987 | 2014 | China | NSP2 482aa 534-562aa,NSP2 630-749aa, GP5a 47-51aa | - |
| 177 | 07HEBTJ | FJ393458 | 2007 | China | NSP2 482aa 534-562aa,GP5a 47-51aa | - |
| 178 | 07NM | FJ393456 | 2007 | China | NSP2 482aa 534-562aa,GP5a 47-51aa | - |
| 179 | NM1 | EU860249 | 2007 | China | NSP2 482aa 534-562aa,GP5a 47-51aa | - |
| 180 | 07HEN | FJ393457 | 2007 | China | NSP2 482aa 534-562aa,GP5a 47-51aa | - |
| 181 | BJSD | FJ950747 | 2007 | China | NSP2 482aa 534-562aa,GP5a 47-51aa | - |
| 182 | SD16 | JX087437 | 2012 | China | NSP2 482aa 534-562aa,GP5a 47-51aa | - |
| 183 | TA-12 | HQ416720 | 2008 | China | NSP2 482aa 534-562aa,GP5a 47-51aa | - |
| 184 | 09SD | JF268678 | 2009 | China | NSP2 482aa 534-562aa,GP5a 47-51aa | - |
| 185 | 09BJ | JF268676 | 2009 | China | NSP2 482aa 534-562aa,GP5a 47-51aa | - |
| 186 | 09HUB5 | GU168568 | 2009 | China | NSP2 482aa 534-562aa,GP5a 47-51aa | - |
| 187 | ZP-1 | HM016159 | 2009 | China | NSP2 482aa 534-562aa,GP5a 47-51aa | - |
| 188 | 09HUB1 | JF268682 | 2009 | China | NSP2 472-501aa 534-562aa, GP5a 47-51aa | - |
| 189 | 09HUB2 | JF268683 | 2009 | China | NSP2 472-501aa 534-562aa, GP5a 47-51aa | - |
| 190 | 09SC | JF268672 | 2009 | China | NSP2 482aa 534-562aa,GP5a 47-51aa | - |
| 191 | SX2009 | FJ895329 | 2009 | China | NSP2 482aa 534-562aa,GP5a 47-51aa | - |
| 192 | WUH3 | HM853673 | 2008 | China | NSP2 482aa 534-562aa,GP5a 47-51aa | - |
| 193 | WUH2 | EU678352 | 2008 | China | NSP2 482aa 534-562aa,GP5a 47-51aa | - |
| 194 | 09HUB7 | GU168567 | 2009 | China | NSP2 482aa 534-562aa,GP5a 47-51aa | - |
| 195 | SC2012 | KM189443 | 2012 | China | NSP2 482aa 486-499aa 534-562aa, GP5a 47-51aa | - |
| 196 | HLJHL | HM189676 | 2009 | China | NSP2 482aa 534-562aa,GP5a 47-51aa | - |
| 197 | 10-10JL | JQ663554 | 2010 | China | NSP2 482aa 534-562aa,GP5a 47-51aa | - |
| 198 | 09DB1 | JF268677 | 2009 | China | NSP2 482aa 534-562aa,GP5a 47-51aa | - |
| 199 | 09DB2 | JF268681 | 2009 | China | NSP2 482aa 534-562aa,GP5a 47-51aa | - |
| 200 | NMG2014 | KM000066 | 2014 | China | NSP2 482aa 534-562aa,GP5a 47-51aa | - |
| 201 | HeN1502 | MF766473 | 2015 | China | NSP2 482aa 534-562aa,GP5a 47-51aa | - |
| 202 | HeNan-A1 | KJ002451 | 2013 | China | NSP2 482aa 534-562aa,GP5a 47-51aa | - |
| 203 | HeN1201 | MF689000 | 2012 | China | NSP2 482aa 534-562aa,GP5a 47-51aa | - |
| 204 | HuN4-F112 | - | - | China | NSP2 482aa 534-562aa,GP5a 47-51aa | - |
| 205 | SDA2 | JX878379 | 2011 | China | NSP2 482aa 534-562aa,GP5a 47-51aa | - |
| 206 | SDA3 | JX878380 | 2011 | China | NSP2 482aa 534-562aa,GP5a 47-51aa | - |
| 207 | NVDC-SD4-2014 | KP771784 | 2014 | China | NSP2 482aa 534-562aa,GP5a 47-51aa | - |
| 208 | Henan-A3 | KJ019330 | 2013 | China | NSP2 482aa 534-562aa,GP5a 47-51aa | - |
| 209 | Henan-A4 | KJ534539 | 2013 | China | NSP2 482aa 534-562aa,GP5a 47-51aa | - |
| 210 | Henan-A13 | KJ819935 | 2014 | China | NSP2 482aa 534-562aa,GP2 247-256aa,GP5a 47-51aa | - |
| 211 | Henan-A12 | KJ819934 | 2014 | China | NSP2 482aa 534-562aa,GP2 247-256aa,GP5a 47-51aa | - |
| 212 | MY-486 | KJ609516 | 2013 | China | NSP2 482aa 534-562aa,GP2 247-256aa,GP5a 47-51aa | - |
| 213 | HLJA1 | KT351739 | 2013 | China | NSP2 474-522aa 534-562aa, GP5a 47-51aa | - |
| 214 | HeN1501 | MF766472 | 2015 | China | NSP2 482aa 534-562aa, GP5a 47-51aa | - |
| 215 | HeNan-A2 | KJ002452 | 2013 | China | NSP2 482aa 534-562aa, GP5a 47-51aa | - |
| 216 | Henan-A14 | KJ819936 | 2014 | China | NSP2 474-522aa 534-562aa,GP2 247-256aa,GP5a 47-51aa | - |
| 217 | HeNan-A9 | KJ546412 | 2013 | China | NSP2 482aa 534-562aa,GP5a 47-51aa | - |
| 218 | MY-376 | KJ609517 | 2013 | China | NSP2 482aa 534-562aa,GP2 247-256aa,GP5a 47-51aa | - |
| 219 | JXja15 | KR149645 | 2015 | China | NSP2 482aa 534-562aa,GP5a 47-51aa | - |
| 220 | HEB 20130008-13 | KP771753 | 2013 | China | NSP2 482aa 534-562aa,GP5a 47-51aa | - |
| 221 | KP | GU232735 | 2008 | China | NSP2 23-43aa 482aa 534-562aa,GP5a 47-51aa | - |
| 222 | HB2014001 | KM261784 | 2014 | China | NSP2 482aa 534-562aa,GP5a 47-51aa | - |
| 223 | JL-0412 | JX177644 | 2012 | China | NSP2 482aa 534-562aa,GP5a 47-51aa | - |
| 224 | NT1 | KP179402 | 2012 | China | NSP2 482aa 534-562aa,GP5a 47-51aa | - |
| 225 | NVDC-SD1-2014 | KP771738 | 2014 | China | NSP2 482aa 534-562aa,GP5a 47-51aa | - |
| 226 | NVDC-SDXX-2013 | KP771741 | 2013 | China | NSP2 482aa 534-562aa,GP5a 47-51aa | - |
| 227 | 11SH1-GD | JX235366 | 2011 | China | NSP2 482aa 534-562aa,GP5a 47-51aa | - |
| 228 | HENZK-1 | KU950373 | 2014 | China | NSP2 482aa 534-562aa,GP5a 47-51aa | - |
| 229 | NVDC-HBCZ-2013 | KP771742 | 2013 | China | NSP2 482aa 534-562aa,GP5a 47-51aa | - |
| 230 | NVDC-MD1-2013 | KP771751 | 2013 | China | NSP2 482aa 534-562aa,GP5a 47-51aa | - |
| 231 | 11SH-GD | JX235365 | 2011 | China | NSP2 482aa 534-562aa,GP5a 47-51aa | - |
| 232 | HNP5 | KT445876 | 2014 | China | NSP2 482aa 534-562aa,GP5a 47-51aa | - |
| 233 | HUN-2014 | KP330232 | 2014 | China | NSP2 482aa 534-562aa,GP5a 47-51aa | - |
| 234 | NVDC-BJ3-2012 | KP771762 | 2012 | China | NSP2 482aa 534-562aa,GP5a 47-51aa | - |
| 235 | JXA1-P80 | FJ548853 | - | China | NSP2 482aa 534-562aa,GP5a 51aa | GP5a 3-6aa |
| 236 | NT2 | KP179403 | 2012 | China | NSP2 482aa 534-562aa,GP5a 47-51aa | - |
| 237 | HENPDS-2 | KU950370 | 2015 | China | NSP2 482aa 534-562aa,GP5a 47-51aa | - |
| 238 | HEB 20130008-14 | KP771752 | 2013 | China | NSP2 482aa 534-562aa,GP5a 47-51aa | - |
| 239 | NVDC-HeB1-2011 | KP771749 | 2011 | China | NSP2 482aa 534-562aa,GP5a 47-51aa | - |
| 240 | NVDC-JS2-2011 | JQ715698 | 2011 | China | NSP2 482aa 495-513aa, GP5a 47-51aa | - |
| 241 | NVDC-MD2-2013 | KP771750 | 2013 | China | NSP2 482aa 534-562aa,GP5a 47-51aa | - |
| 242 | HEB-2013 | KJ591659 | 2013 | China | NSP2 482aa 534-562aa,GP5a 47-51aa | - |
| 243 | NT3 | KP179404 | 2012 | China | NSP2 482aa 498-499aa 534-562aa, GP5a 47-51aa | - |
| 244 | Shaanxi-2 | HQ401282 | 2007 | China | NSP2 482aa 534-562aa,GP5a 47-51aa | - |
| 245 | HNyc13 | KT022072 | 2013 | China | NSP2 482aa 534-562aa,GP5a 47-51aa | - |
| 246 | NVDC-BJPG-2013 | KP771743 | 2013 | China | NSP2 482aa 534-562aa,GP5a 47-51aa | - |
| 247 | YD | JF748717 | 2009 | China | NSP2 482aa 490-511 534-562aa, GP5a 47-51aa | - |
| 248 | GZgy15-1 | KT358728 | 2015 | China | NSP2 482aa 488-516aa 534-562aa, GP5a 47-51aa | - |
| 249 | NVDC-SD6-2014 | KP771737 | 2014 | China | NSP2 482aa 534-562aa,GP5a 47-51aa | - |
| 250 | 11NZ-GD | JX217036 | 2011 | China | NSP2 482aa 534-562aa,GP5a 47-51aa | - |
| 251 | 11XX-GD | JX235367 | 2011 | China | NSP2 482aa 534-562aa,GP5a 47-51aa | - |
| 252 | GX1001 | JQ955657 | 2011 | China | NSP1 39aa, NSP2 482aa 534-562aa, ORF1b 14aa, GP2 14aa, GP3 5aa, GP4 6aa, GP5 3aa, GP5a 47-51aa | NSP9 478-480aa，GP2 259-261 |
| 253 | GX1003 | JX912249 | 2010 | China | NSP2 482aa 534-562aa,GP5a 47-51aa | - |
| 254 | NVDC-shh01-2014 | KP771736 | 2014 | China | NSP2 482aa 534-562aa,GP5a 47-51aa | - |
| 255 | NVDC-SHH02-2014 | KP771735 | 2014 | China | NSP2 482aa 534-562aa,GP5a 47-51aa | - |
| 256 | NVDC-BJ9-2012 | KP771756 | 2012 | China | NSP2 482aa 534-562aa,GP5a 47-51aa | - |
| 257 | NVDC-SD2-2012 | KP771768 | 2012 | China | NSP2 482aa 534-562aa,GP5a 47-51aa | - |
| 258 | NVDC-13SXJC-2014 | KP771780 | 2014 | China | NSP2 482aa 534-562aa,GP5a 47-51aa | - |
| 259 | NVDC-SXJC-2013 | KP771740 | 2013 | China | NSP2 482aa 534-562aa,GP5a 47-51aa | - |
| 260 | HNxa14 | KT022071 | 2014 | China | NSP2 482aa 534-562aa,GP5a 47-51aa | - |
| 261 | GX1002 | JQ955658 | 2010 | China | NSP2 482aa 534-562aa,GP5a 47-51aa | - |
| 262 | HB-XL | KP162169 | 2014 | China | NSP2 482aa 534-562aa,GP5a 47-51aa | - |
| 263 | NVDC-SD1-2012 | KP771769 | 2012 | China | NSP2 482aa 534-562aa,GP5a 47-51aa | - |
| 264 | NVDC-BJ4-2012 | KP771761 | 2012 | China | NSP2 482aa 534-562aa,GP5a 47-51aa | - |
| 265 | NVDC-BJ5-2012 | KP771760 | 2012 | China | NSP2 482aa 534-562aa,GP5a 47-51aa | - |
| 266 | 10BY-GD | JX192636 | 2010 | China | NSP2 482aa 534-562aa,GP5a 47-51aa | - |
| 267 | NVDC-HeB1-2012 | KP771773 | 2012 | China | NSP2 482aa 534-562aa,GP5a 47-51aa | - |
| 268 | BJ1102 | KF751237 | 2011 | China | NSP2 482aa 534-562aa,GP5a 47-51aa | - |
| 269 | 10FS-GD | JX192634 | 2010 | China | NSP2 482aa 534-562aa,GP5a 47-51aa | - |
| 270 | NVDC-GD-2011 | KP771766 | 2011 | China | NSP2 482aa 534-562aa,GP5a 47-51aa | - |
| 271 | 10ZQ-GD | JX192639 | 2010 | China | NSP2 482aa 534-562aa,GP5a 47-51aa | - |
| 272 | 10-10FUJ-2 | JQ663547 | 2010 | China | NSP2 482aa 534-562aa,GP5a 47-51aa | - |
| 273 | XF1129 | KT180169 | 2013 | China | NSP2 482-517aa 534-562aa,GP5a 47-51aa | - |
| 274 | NVDC-BJ1-2012 | KP771764 | 2012 | China | NSP2 482aa 534-562aa,GP5a 47-51aa | - |
| 275 | NVDC-BJ2-2012 | KP771763 | 2012 | China | NSP2 482aa 534-562aa,GP5a 47-51aa | - |
| 276 | 10-10HEB-3 | JQ663553 | 2010 | China | NSP2 482aa 534-562aa,GP5a 47-51aa | - |
| 277 | GD-2011 | KC527830 | 2011 | China | NSP2 482aa 534-562aa,GP5a 47-51aa | GP5 60aa |
| 278 | 09HEN1 | JF268684 | 2009 | China | NSP2 482aa 534-562aa,GP5a 47-51aa | - |
| 279 | NVDC-HeB2-2011 | KP771765 | 2011 | China | NSP2 482aa 534-562aa,GP5a 47-51aa | - |
| 280 | Shanxi-6 | KJ855518 | 2010 | China | NSP2 482aa 534-562aa,GP5a 47-51aa | - |
| 281 | 10-10GX-2 | JQ663559 | 2010 | China | NSP2 482aa 534-562aa,GP5a 47-51aa | - |
| 282 | 10-10GX-4 | JQ663561 | 2010 | China | NSP2 482aa 534-562aa,GP5a 47-51aa | - |
| 283 | 10QY-GD | JX215552 | 2010 | China | NSP2 482aa 534-562aa,GP5a 47-51aa | - |
| 284 | 10SJ-GD | JX192637 | 2010 | China | NSP2 482aa 534-562aa,GP5a 47-51aa | - |
| 285 | 10FS1-GD | JX192635 | 2010 | China | NSP2 482aa 534-562aa,GP5a 47-51aa | - |
| 286 | 10HD-GD | JX215553 | 2010 | China | NSP2 482aa 534-562aa,GP5a 47-51aa | - |
| 287 | 10SS-GD | JX192638 | 2010 | China | NSP2 482aa 534-562aa,GP5a 47-51aa | - |
| 288 | 10-10JX | JQ663540 | 2010 | China | NSP2 482aa 534-562aa,GP5a 47-51aa | - |
| 289 | 09HEN2 | JF268680 | 2009 | China | NSP2 482aa 534-562aa,GP5a 47-51aa | - |
| 290 | FS | JF796180 | 2010 | China | NSP2 482aa 534-562aa,GP5a 47-51aa | - |
| 291 | NVDC-BJ1-2011 | KP771778 | 2011 | China | NSP2 482aa 534-562aa,GP5a 47-51aa | - |
| 292 | 10-10BJ-3 | JQ663542 | 2010 | China | NSP2 482aa 534-562aa,GP5a 47-51aa | - |
| 293 | 10-10BJ-4 | JQ663544 | 2010 | China | NSP2 482aa 534-562aa,GP5a 47-51aa | - |
| 294 | 10-10FUJ-3 | JQ663548 | 2010 | China | NSP2 482aa 534-562aa,GP5a 47-51aa | - |
| 295 | 10-10FUJ-4 | JQ663549 | 2010 | China | NSP2 482aa 534-562aa,GP5a 47-51aa | - |
| 296 | NVDC-HeN-2012 | KP771771 | 2012 | China | NSP2 482aa 534-562aa,GP5a 47-51aa | - |
| 297 | NVDC-HuN-2011 | KP771770 | 2012 | China | NSP2 482aa 534-562aa,GP5a 47-51aa | - |
| 298 | 10-10GX-3 | JQ663560 | 2010 | China | NSP2 482aa 534-562aa,GP5a 47-51aa | - |
| 299 | 10-10BJ-2 | JQ663543 | 2010 | China | NSP2 482aa 534-562aa,GP5a 47-51aa | - |
| 300 | 10-10HEB-2 | JQ663552 | 2010 | China | NSP2 482aa 534-562aa,GP5a 47-51aa | - |
| 301 | 09JS | JF268675 | 2009 | China | NSP2 482aa 534-562aa,GP5a 47-51aa | - |
| 302 | BB0907 | HQ315835 | 2009 | China | NSP2 482aa 534-562aa,GP5a 47-51aa | - |
| 303 | JN-HS | HM016158 | 2008 | China | NSP2 482aa 534-562aa,GP5a 47-51aa | - |
| 304 | 10-10FUJ-5 | JQ663550 | 2010 | China | NSP2 482aa 534-562aa,GP5a 47-51aa | - |
| 305 | 10-10SD | JQ663555 | 2010 | China | NSP2 482aa 534-562aa,GP5a 47-51aa | - |
| 306 | 10-10BJ-1 | JQ663541 | 2010 | China | NSP2 482aa 534-562aa,GP5a 47-51aa | - |
| 307 | 10-10BJ-5 | JQ663545 | 2010 | China | NSP2 482aa 534-562aa,GP5a 47-51aa | - |
| 308 | 10-10GX-1 | JQ663558 | 2010 | China | NSP2 482aa 534-562aa | - |
| 309 | DC | JF748718 | 2010 | China | NSP2 154aa 482aa 534-562aa, GP5a 47-51aa | - |
| 310 | 10-10GX-5 | JQ663562 | 2010 | China | NSP2 482aa 534-562aa,GP5a 47-51aa | - |
| 311 | 10-10FUJ-1 | JQ663546 | 2010 | China | NSP2 482aa 534-562aa,GP5a 47-51aa | - |
| 312 | 10-10HEB-1 | JQ663551 | 2010 | China | NSP2 482aa 534-562aa,GP5a 47-51aa | - |
| 313 | NVDC-YN-2011 | KP771767 | 2011 | China | NSP2 482aa 534-562aa,GP5a 47-51aa | - |
| 314 | 10-LW3-7 | JQ663564 | 2010 | China | NSP2 482aa 534-562aa,GP5a 47-51aa | - |
| 315 | 10-10LW5-1 | JQ663565 | 2010 | China | NSP2 482aa 534-562aa,GP5a 47-51aa | - |
| 316 | 10-LW6-6 | JQ663566 | 2010 | China | NSP2 482aa 534-562aa,GP5a 47-51aa | - |
| 317 | 10-LW1-13 | JQ663557 | 2010 | China | NSP2 482aa 534-562aa,GP5a 47-51aa | - |
| 318 | 10-LW2-6 | JQ663563 | 2010 | China | NSP2 482aa 534-562aa,GP5a 47-51aa | - |
| 319 | 10-LW7-1 | JQ663567 | 2010 | China | NSP2 482aa 534-562aa,GP5a 47-51aa | - |
| 320 | 10-LW8-1 | JQ663568 | 2010 | China | NSP2 482aa 534-562aa,GP5a 47-51aa | - |
| 321 | NADC30 | JN654459 | 2008 | USA | NSP2 324-434aa 486aa 502-520aa, GP5a 47-51aa | - |
| 322 | FJLIUY-2017 | MG011718 | 2017 | China | NSP2 324-434aa 486aa 502-520aa,GP5a 47-51aa | - |
| 323 | SCcd17 | MG914067 | 2017 | China | NSP2 324-434aa 486aa 502-520aa,GP5a 39aa 47-51aa, GP5 37aa | - |
| 324 | CY1-1604 | MH651736 | 2016 | China | NSP2 324-434aa 486aa 502-520aa,GP5a 47-51aa | - |
| 325 | JL580 | KR706343 | 2013 | China | NSP2 324-434aa 486aa 502-520aa,GP5a 47-51aa | - |
| 326 | SDYG1606 | KY053458 | 2016 | China | NSP2 324-434aa 486aa 502-520aa,GP5a 47-51aa | - |
| 327 | SD-1602 | MH651743 | 2016 | China | NSP2 324-434aa 486aa 502-520aa 805-806aa,GP5a 47-51aa | - |
| 328 | HENZMD-9 | KU950374 | 2015 | China | NSP2 324-434aa 486aa 502-520aa,GP5a 47-51aa | - |
| 329 | SC-d | MF375261 | 2015 | China | NSP2 324-434aa 486aa 502-520aa 805-806aa,GP5a 47-51aa | - |
| 330 | SCN17 | MH078490 | 2017 | China | NSP2 324-434aa 486aa 502-520aa,GP5a 39aa 47-51aa, GP5 37aa | - |
| 331 | FJL15 | KY412887 | 2014 | China | NSP2 324-434aa 486aa 502-520aa,GP5a 47-51aa | - |
| 332 | SDQD-1604 | MH651742 | 2016 | China | NSP2 324-434aa 486aa 502-520aa,GP5a 47-51aa | - |
| 333 | HBFL-1604 | MH651739 | 2016 | China | NSP2 16-17aa 324-434aa 486aa 502-520aa, GP5a 47-51aa | - |
| 334 | FJDJQ-2017 | MG011719 | 2017 | China | NSP2 324-434aa 486aa 502-520aa | - |
| 335 | HNJYH-1606 | MH651740 | 2016 | China | NSP2 324-434aa 486aa 502-520aa,GP5a 47-51aa | - |
| 336 | HENAN-HEB | KJ143621 | 2012 | China | NSP2 324-434aa 486aa 502-520aa,GP5a 47-51aa | - |
| 337 | HENAN-XINX | KF611905 | 2013 | China | NSP2 324-434aa 486aa 502-520aa,GP5a 47-51aa | - |
| 338 | SDbz16-2 | MH588710 | 2016 | China | NSP2 324-434aa 486aa 502-520aa,GP5a 47-51aa | - |
| 339 | HNJYF-1606 | MH651738 | 2016 | China | NSP2 324-434aa 486aa 502-520aa,GP5a 47-51aa | - |
| 340 | HNyc15 | KT945018 | 2015 | China | NSP2 324-434aa 486aa 502-520aa | - |
| 341 | SD17-38 | MH068878 | 2017 | China | NSP2 324-434aa 486aa 502-520aa,GP5a 39aa 47-51aa,GP5 37aa | GP5 58aa |
| 342 | SDZC-1609 | MH651747 | 2016 | China | NSP2 324-434aa 486aa 502-520aa,GP5a 47-51aa | - |
| 343 | CHsx1401 | Kp861625 | 2014 | China | NSP2 324-434aa 486aa 502-520aa,GP5a 47-51aa | - |
| 344 | WUH6 | KU523367 | 2011 | China | NSP2 324-434aa 486aa 502-520aa,GP5a 47-51aa | - |
| 345 | NADC30 | MH500776 | 2017 | China | NSP2 324-434aa 486aa 502-520aa,GP5a 39aa 47-51aa,GP5 37aa | - |
| 346 | SD-A19 | MF375260 | 2015 | China | NSP2 324-434aa 486aa 502-520aa,GP5a 39aa 47-51aa,GP5 37aa | - |
| 347 | HB17A | MG844181 | 2017 | China | NSP2 324-434aa 486aa 502-520aa 585-586aa,GP5a 47-51aa | - |
| 348 | TJZH-1607 | MH651748 | 2016 | China | NSP2 324-434aa 486aa 502-520aa 585-586aa,GP5a 47-51aa | - |
| 349 | QHD3 | MH167388 | 2017 | China | NSP2 324-434aa 466-470aa 486aa 502-520aa 586-587aa,GP5a 47-51aa | - |
| 350 | LNCH-1604 | MH651741 | 2016 | China | NSP2 324-434aa 486aa 502-520aa,GP5a 47-51aa | - |
| 351 | QHD2 | MH167387 | 2017 | China | NSP2 324-434aa 486aa 502-520aa,GP5a 47-51aa | - |
| 352 | HENXX-1 | KU950372 | 2014 | China | NSP2 324-434aa 486aa 502-520aa,GP5a 47-51aa | - |
| 353 | HNjz15 | KT945017 | 2015 | China | NSP2 324-434aa 486aa 502-520aa,GP2 256aa,GP5a 47-51aa | - |
| 354 | HENXC-4 | KU950371 | 2015 | China | NSP2 324-434aa 486aa 502-520aa,GP5a 47-51aa | GP5 58aa |
| 355 | WUH5 | KU523366 | 2015 | China | NSP2 324-434aa 486aa 502-520aa,GP5a 47-51aa | - |
| 356 | CY2-1604 | MH651737 | 2016 | China | NSP2 324-434aa 486aa 502-520aa,GP5a 39aa 47-51aa, GP5 37aa | - |
| 357 | SD99-1606 | MH651745 | 2016 | China | NSP2 324-434aa 486aa 502-520aa,GP5a 39aa 47-51aa, GP5 37aa | - |
| 358 | SD17-36 | MH121061 | 2017 | China | NSP2 324-434aa 486aa 502-520aa,NSP7 211aa, GP5a 39aa, GP5 37aa | - |
| 359 | SDQZ-1609 | MH651746 | 2016 | China | NSP2 324-434aa 486aa 502-520aa,GP5a 39aa 47-51aa, GP5 37aa | - |
| 360 | QHD1 | MG687491 | 2017 | China | NSP2 324-434aa 486aa 502-520aa,GP5a 39aa, GP5 37aa | - |
| 361 | SD53-1603 | MH651744 | 2016 | China | NSP2 324-434aa 486aa 502-520aa,GP5a 39aa 47-51aa, GP5 37aa | - |
| 362 | MN184A | DQ176019 | 2002 | USA | NSP2 324-434aa 486aa 502-520aa,GP5a 47-51aa | - |
| 363 | Minnesota1 | KP283414 | 2012 | USA | NSP2 324-434aa 486aa 502-520aa,GP5a 47-51aa | - |
| 364 | LNWK130 | MG913987 | 2017 | China | NSP2 330-429aa,GP5a 47-51aa | - |
| 365 | LNWK96 | MG860516 | 2017 | China | NSP2 330-429aa,GP5a 47-51aa | - |
